# Protistan Plankton Responses to Variable Light and Upwelling in the Peruvian Humboldt Current System: Insights Into Community Dynamics Under Environmental Change

**DOI:** 10.1002/ece3.72827

**Published:** 2026-01-12

**Authors:** Sven Katzenmeier, Megan Gross, Thorsten Stoeck

**Affiliations:** ^1^ Ecology Group University of Kaiserslautern‐Landau (RPTU) Kaiserslautern Germany

**Keywords:** Humboldt upwelling system, mesocosm experiments, metabarcoding, Protistan plankton, time series

## Abstract

The Humboldt Current System (HCS) sustains one of the most productive fisheries globally, driven by nutrient‐rich upwelling. Understanding the impact of climate change, including variations in upwelling intensities and light availability, on protistan plankton communities is critical due to their pivotal role in food web dynamics and biogeochemical cycles. During the CUSCO campaign, eight off‐shore mesocosms were deployed to simulate varying upwelling intensities (0%, 15%, 30%, and 45% deep‐water addition) under high‐light (HL) and low‐light (LL) scenarios representing austral summer and winter conditions. DNA metabarcoding targeting the V9 18S rRNA gene revealed significant changes in diversity, community structure, and functional roles. Increased upwelling enhanced alpha diversity, likely reflecting a combination of nutrient enrichment and dispersal of deep‐water protists, with diatoms and chlorophytes dominating under nutrient‐enriched and depleted conditions, respectively. In contrast, dinoflagellates thrived under high‐light, low‐upwelling conditions, showing a functional shift toward parasitism and osmotrophic lifestyle under nutrient deficiency. These findings underscore the potential impacts of climate‐induced changes on protistan plankton diversity, food web structures, and ecosystem services in upwelling systems.

## Graphical abstract

Our study reveals (based on experiments) the shifts in protistan plankton communities, serving as energy and organic matter source for higher trophic levels if the Humboldt Current System is affected by climate change as predicted by current models. This may have profound effects on fisheries in the Humboldt Current System.

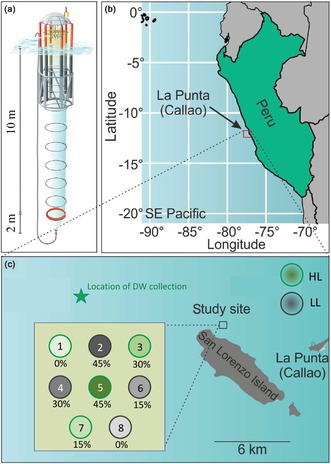

## 1 Introduction

Coastal upwelling systems, particularly Eastern Boundary Upwelling Systems (EBUS), are renowned for their high productivity despite covering less than 1% of the global ocean area (Ryther 1969; Chavez and Messié 2009; Cushing 1971; Pauly and Christensen 1995). The Humboldt Current System (HCS) off the coast of Peru is a paradigmatic example in which the transport of nutrient‐rich deep water (DW) by equatorward cold currents sustains remarkably high primary production and supports some of the world's largest fisheries (Chavez and Messié 2009; Bakun and Nelson 1991). Protistan plankton play a central role in these ecosystems by driving primary production, nutrient recycling, and energy transfer via the microbial loop—a concept established in the mid‐1970s (Azam et al. 1983; Cotner and Biddanda 2002; Field et al. 1998; Caron et al. 1988).

Despite their ecological importance, our mechanistic understanding of how protistan communities respond to environmental variability remains limited. Previous studies have largely focused on the influence of nutrient enrichment and light limitation on phytoplankton or bacterioplankton (Browning and Moore 2023; Edwards et al. 2016; Landry and Hassett 1982; Redfield 1958). However, the combined effects of altered upwelling intensity and shifting light regimes on protistan assemblages in dynamic coastal environments are less well understood (Messié and Chavez 2015; Gruber et al. 2011). This gap is particularly critical given that protists often mediate the ecological consequences of environmental change by restructuring microbial interactions and modifying nutrient regeneration pathways. Climate change is expected to modify both the magnitude of upwelling events and the coastal light environment through changes in cloud cover, water turbidity, and circulation patterns (Doney et al. 2012; Seitzinger et al. 2010), yet how these drivers interact to shape protistan diversity and trophic function remains an open question.

Recent projections suggest that anthropogenic climate change will have profound effects on the physical forcing in the HCS. Bakun et al. (2015) and Messié and Chavez (2015) predict that altered coastal winds and modified atmospheric circulation will lead to changes in the timing and intensity of upwelling events, potentially reducing the nutrient supply in some areas while shifting the seasonal dynamics of the system. In parallel, changes in precipitation patterns, sea surface temperatures, and cloud cover are expected to modify the underwater light field, thereby influencing the growth rates, photosynthetic capacity, and metabolic activity of marine organisms (Doney et al. 2012; Seitzinger et al. 2010). Because both nutrient delivery and irradiance regime exert primary control over protistan physiology, alterations in these drivers could restructure community composition, disrupt biogeochemical cycles, and propagate upward to influence higher trophic levels and fisheries productivity. These anticipated changes underscore the need to understand the combined impact of altered upwelling and light availability on protistan communities, which are pivotal in mediating ecosystem processes and supporting higher trophic levels. Yet empirical data that jointly manipulate these drivers in situ remain scarce, limiting our ability to forecast ecological tipping points and shifts in productivity regimes.

Here, we address these gaps by conducting an in situ mesocosm experiment in the HCS that simultaneously manipulates upwelling intensity and light availability. In our experimental design, deep, nutrient‐rich water was injected in varying proportions (0%, 15%, 30%, and 45%) into mesocosms, while light conditions were controlled to simulate austral summer (high light) and austral winter (low‐light) scenarios (Xue et al. 2022). DNA metabarcoding of the V9 region of the 18S rRNA gene (Stoeck et al. 2010; Bach et al. 2020; Min et al. 2023) was used to characterize temporal changes in both the taxonomic composition and functional traits of the protistan communities. Specifically, we test whether the direct input of DW and its associated immigrant plankton enhances local diversity and drives shifts in trophic strategies, and how these effects are modulated by light availability. Our experimental design therefore simulates a realistic upwelling pulse by substituting a portion of mesocosm water with DW of differing intensities. This approach reflects the natural coupling of nutrient injection and microbial dispersal that occurs during physical upwelling events. While these two processes cannot be fully disentangled within our setup, their combined effects provide insight into how protistan communities respond to abrupt changes in resource availability and microbial mixing. To guide our investigation, we formulated two hypotheses: (H1) Light availability was expected to structure protistan communities by constraining phototrophic pathways, leading to a relative increase in heterotrophic and mixotrophic taxa under reduced irradiance. (H2) Upwelling intensity was anticipated to alter community composition through the combined effects of nutrient enrichment and the dispersal of DW microbial taxa, resulting in temporary increases in alpha diversity and shifts toward taxa adapted to nutrient‐rich conditions.

By integrating controlled field experiments with advanced molecular techniques and rigorous statistical analyses, our study provides novel insights into the ecological interactions underpinning protistan community dynamics in a highly productive upwelling system. These findings are critical for predicting how climate‐driven alterations in environmental forcing may cascade through microbial food webs, alter biogeochemical cycles, and ultimately affect ecosystem services such as fisheries and carbon export.

## 2 Materials and Methods

**FIGURE 1:**
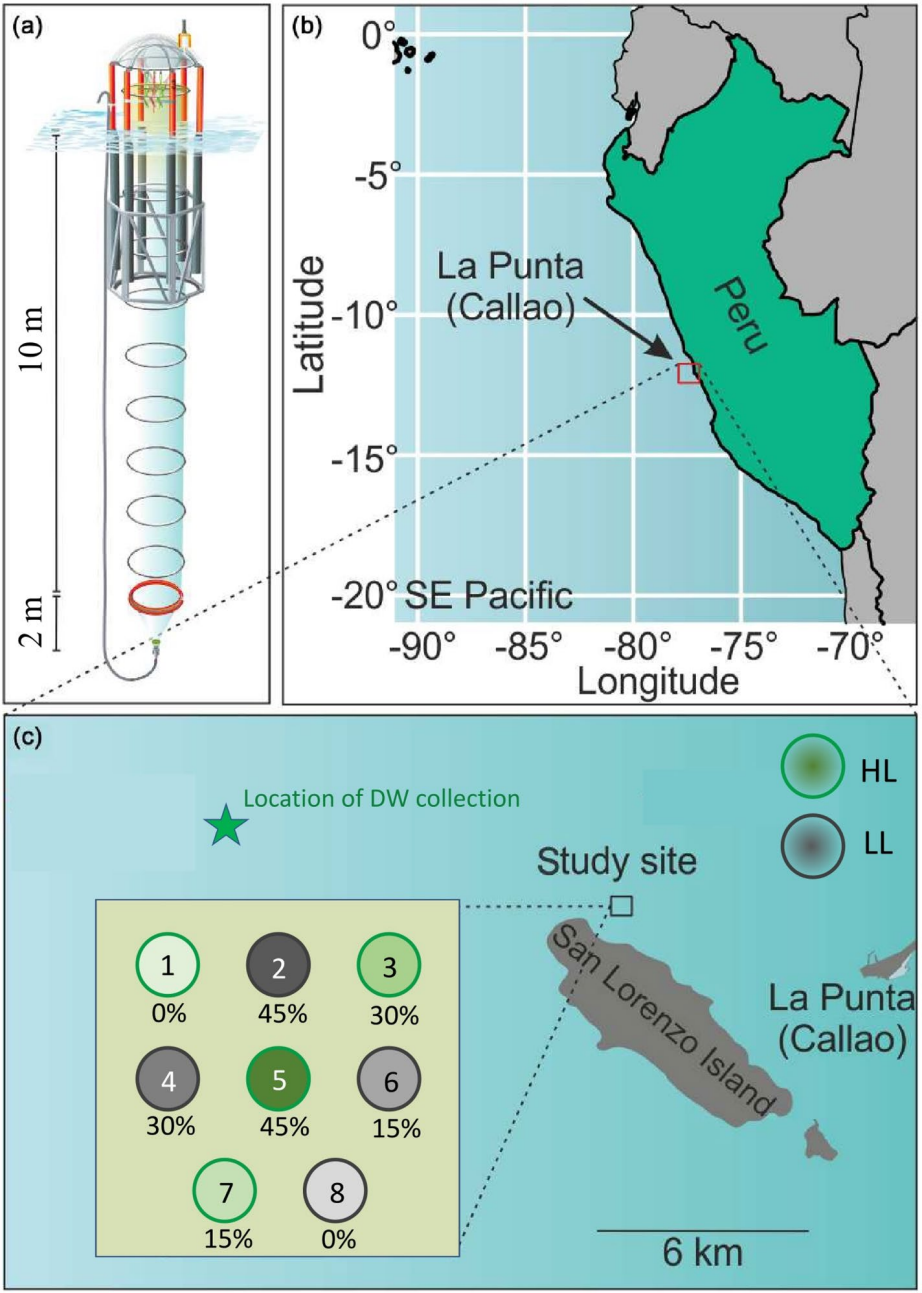
Experimental design and study location. (a) Dimensions of one mesocosm with an underwater bag. Please note the size ratio for the underwater bag is not to scale. (b) Large‐scale map of the study site in the Humboldt Current System. (c) Detailed map of the study area and the mesocosm arrangement for the light and upwelling manipulation. The star marks the location where the collection of the deep water (DW) occurred. The percentages indicate the volume of water exchanged with DW for each mesocosm (see Table 1 for time of deep‐water treatment). This corresponds to the darkening of the color. The high light (HL), as no manipulation, and low light (LL) to simulate austral winter are colored green and gray, respectively. Modified from Bach et al. (2020).

An assessment of the impact of upwelling intensity and light limitation on protistan plankton communities was carried out in the southeast Pacific. The experimental setup was established approximately 6 km off the coast of La Punta (Callao, Peru), in the north‐eastern vicinity of the Isle of San Lorenzo (12.06° S, 77.24° W; Figure 1) during the austral summer of 2020, spanning from February 28 to April 3. Nine Kiel Off‐Shore Mesocosms for Future Ocean Simulations (KOSMOS M1‐M9; Riebesell et al. 2013) were deployed and anchored on February 19, 2020, using the research vessels BIC Humboldt and IMARPE VI. These mesocosms consisted of underwater bags, measuring 12 m in length and 2 m in diameter, constructed from polyurethane foil secured to flotation frames. A comprehensive description of the mesocosms can be found in Riebesell et al. (2013).

To prevent interference from birds, the flotation frames were equipped with covers and spikes designed to deter them from perching and contaminating the experimental setup. The underwater bags were submerged and enclosed with nets featuring a 3‐mm mesh size at both their top and bottom openings, allowing for water exchange with the open Pacific while restricting the entry of larger organisms. Due to logistical constraints, sampling from M9 was excluded from this study and is therefore not considered further.

The experiment formally commenced on February 28, 2020 (day 0), with the enclosure of the underwater bags. The bottom nets were replaced with sediment traps, and the bags were elevated approximately 1.5 m above the sea surface to prevent mixing with surrounding waters. Additionally, larger organisms such as jellyfish, larvae, and fish were removed using a 1‐mm mesh‐sized net.

To minimize biofouling within the mesocosms, divers routinely cleaned the external walls of the underwater bags, while the interior surfaces were maintained using a specially designed ring‐shaped double‐bladed wiper, as described in Bach et al. (2016) and Riebesell et al. (2013).

### 2.1 Light and Upwelling Simulation

Light limitation was implemented by shading the enclosure bags of four KOSMOS units (M2, M4, M6, M8) with opaque polyurethane foil, hereafter referred to as low‐light (LL) mesocosms. The high‐light (HL) KOSMOS units (M1, M3, M5, M7) featured transparent bags, allowing for natural light conditions characteristic of the austral summer. In the HL mesocosms, the average photosynthetically active radiation (PAR), measured using a CTD device (CTD167M, Sea and Sun Technologies), was 173 μmol photons m^−2^ s^−1^. The shading of LL mesocosms reduced light intensity by approximately 50%, resulting in an average PAR of 86 μmol photons m^−2^ s^−1^. The 50% light reduction was selected as an experimental contrast to represent a marked decrease in underwater irradiance comparable to austral winter or increased turbidity conditions, rather than a literal projection of future light attenuation under climate change. While climate change is expected to alter light availability through changes in cloud cover, stratification, and turbidity, its magnitude remains uncertain; our chosen levels therefore serve as controlled scenarios to test community sensitivity to reduced light.

Upwelling simulations were executed by substituting mesocosm water with nutrient‐rich DW. This was carried out using three different upwelling intensities (15%, 30%, and 45%) denoting the percentage volume of water exchanged, alongside a reference mesocosm (0%) without DW addition. This procedure introduces both elevated nutrient concentrations and the DW microbial assemblage into the mesocosms, consistent with natural upwelling events. While nutrient changes after the pulse were quantified, the microbial composition of the DW source was not sequenced, which limits our ability to partition nutrient‐driven responses from dispersal effects. Preparatory steps were essential to simulate upwelling intensities accurately across the KOSMOS units. Initially, the volume of water enclosed in each mesocosm was quantified to calculate the precise amount of water to be replaced with DW. To achieve this, a calibrated NaCl (sodium chloride) solution was homogenously introduced into the water column to induce a minor increase in salinity. Salinity measurements were conducted using CTD devices both before and after the solution's introduction. These measurements, combined with seawater density calculations and the volume of NaCl solution applied, allowed for the determination of enclosed water volume (File S1; Czerny et al. 2013).

Nutrient‐rich DW was collected on March 6, 2020, in alignment with methods described by Bach et al. (2020) and Taucher et al. (2017). Using a DW collector with a capacity of 100 m^3^, the water was sourced from a depth of 40 m along the IMARPE time‐series transect (Graco et al. 2017). Subsequent water exchanges were carried out on March 11 (day 13) for LL mesocosms and March 12 (day 14) for HL mesocosms via a submersible pump (Grundfos SP 17‐5R; pump rate ~18 m^3^ h^−1^). This process established nutrient levels in the manipulated mesocosms that were representative of natural upwelling areas (File S1; Hauschildt et al. 2021; Igarza et al. 2019; Messié et al. 2009).

Additionally, on March 17, fish larvae comprising 120 *Engraulis ringens* (Peruvian anchoveta) and 70 *Anisotremus scapularis* (Peruvian grunt) were introduced into each KOSMOS. These two species were selected for their ecological and economic importance in the HCS: *E. ringens* is an essential forage fish and the basis for one of the largest single‐fisheries, while *A. scapularis* plays a pivotal role in local coastal food webs and artisanal fisheries (Bertrand et al. 2004; Chavez et al. 2008). The addition of fish larvae was intended to simulate natural recruitment and trophic interactions typical of post‐upwelling coastal systems. However, we acknowledge that this biological manipulation introduces additional variability and potential confounding effects when isolating the impacts of light and upwelling on protistan communities.

Rigid‐hulled inflatable boats stationed at the harbor of La Punta were utilized to facilitate sampling at the mesocosm site. These boats were securely moored to the flotation frames to provide a stable platform for sampling procedures. A hose (10 m in length, 0.05 m in diameter) was employed to collect water samples from the entire water column of each mesocosm. Approximately 12 L of water was extracted per mesocosm and transferred into plastic containers. To ensure sample integrity during transport to the laboratory, the containers were placed in tubs filled with seawater to mitigate temperature fluctuations and covered with opaque polyurethane foil to prevent light exposure.

Due to logistical constraints associated with operating large‐scale mesocosm systems (KOSMOS), each combination of light and upwelling treatment was represented by a single mesocosm. Consequently, the experiment was conducted without true replication at the treatment level, and the water samples collected over time from within each mesocosm represent pseudo‐replicates rather than independent experimental units. This limitation is common in large‐scale mesocosm studies due to the substantial technical, financial, and spatial requirements involved (see Riebesell et al. 2013; Bach et al. 2016). Furthermore, only four mesocosms were processed per sampling day. The specific sampling sequence adhered to the schedule detailed in Table 1. Notably, prior to experimental day 21, COVID‐19 regulations posed a significant risk of experiment termination. Consequently, adjustments were made to the schedule on short notice for days 21 and 23 to prioritize data collection that would enable comparisons of light and upwelling conditions, even if day 21 had marked the conclusion of the experiment. However, official authorization from the Peruvian government permitted the continuation of the project, and as of experimental day 27, the original sampling schedule was reinstated.

**TABLE 1:** Sampling procedure and treatments for mesocosms: The sampling schedule was adjusted (days 21/23) during the field campaign to accommodate potential experiment termination due to COVID‐19 regulations.

Date	Experimental day	Mesocosm
02/28/2020	1	HL0; HL15; HL30; HL45
03/01/2020	3	LL0; LL15; LL30; LL45
03/05/2020	7	HL0; HL15; HL30; HL45
03/07/2020	9	LL0; LL15; LL30; LL45
03/11/2020	13	LL‐deep‐water addition
03/12/2020	14	HL‐deep‐water addition
03/13/2020	15	LL0; LL15; LL30; LL45
03/15/2020	17	HL0; HL15; HL30; HL45
03/19/2020	21	HL0; HL45; LL0; LL45
03/21/2020	23	HL15; HL30; LL15; LL30
03/25/2020	27	HL0; HL15; HL30; HL45
03/27/2020	29	LL0; LL15; LL30; LL45
03/31/2020	33	HL0; HL15; HL30; HL45
04/02/2020	36	LL0; LL15; LL30; LL45

Abbreviations: HL, high light; LL, low light.

### 2.2 Physicochemical Parameters

Temperature, oxygen, pH, salinity, chlorophyll a (Chl‐a), and PAR were measured by the CTD sensor system. The data were subsequently averaged for the complete water column. Samples for nutrient measurement were taken by 5 L ‘integrating water samplers’ (Hydro‐Bios, Kiel) over a depth of 0–10 m. Triplicate samples were filtered (0.45 μm pore size, Sterivex, Merck, Darmstadt, DE) and analyzed by a QuAAtro AutoAnalyzer (SEAL Analytical with an XY‐2 Sampler, Mequon, US) to colorimetrically measure phosphate (PO_4_^3−^), silicic acid (Si(OH)_4_), nitrate (NO_3_^−^), nitrite (NO_2_^−^) (Morris and Riley 1963; Mullin and Riley 1955; Murphy and Riley 1962). All physicochemical parameters were determined by the CUSCO 2020 consortium and available in the Supporting information file section “Physico chemical dynamics” and Files S2 and S3.

### 2.3 Filter Preparation

To investigate protistan plankton community composition we applied eDNA‐metabarcoding. Therefore, water samples were filtered immediately after sampling onto 0.65 μm pore‐sized Durapore membrane filters (Millipore, diameter 47 mm) using the rotarus peristaltic pump. Three replicates were taken per mesocosm, each with roughly 3 L of water. This resulted in a total of 144 filter samples. The filters were placed into cryogenic vials and preserved using Qiagen's LifeGuard Soil Preservation Solution. Samples were stored at −20°C and transported to Germany by a reefer until further processing in the laboratory.

### 2.4 DNA Extraction and Amplification

The DNeasy PowerWater Kit (Qiagen) was used to extract the DNA from all three filter replicates according to the manufacturer's protocol. Following Stoeck et al. (2010) the hypervariable V9 region of the 18S rDNA gene was PCR‐amplified in 50 μL‐reactions using the 1391F as forward primer (5′‐GTACACACCGCCCGTC‐3′) (Lane 1991) and the EukB as reverse primer (5′‐TGATCCTTCTGCAGGTTCACCTAC‐3′) (Medlin et al. 1988). The sequential order of the PCR comprised an initial denaturation step (98°C for 30 s), followed by 30 cycles of denaturation (10 s at 98°C), annealing (20 s at 61°C), elongation (25 s at 72°C), and a final five‐minute extension at 72°C. The expected fragment length of approximately 150 base pairs was verified using gel‐electrophoresis, and PCR products were subsequently frozen at −20°C until preparation for high‐throughput sequencing (HTS).

### 2.5 Sequencing, Quality Control, Clustering, and Taxonomic Assignment

The company SeqIT GmbH & Co. KG performed the paired‐end Illumina MiSeq sequencing. The raw data files are publicly available in the Sequence Read Archive of the National Center for Biotechnology Information under project number PRJNA1286811. With CUTADAPT v1.18 (Martin 2011), primers were removed and the FASTX toolkit (RRID: SCR_05534) sorted the sequences in the same read direction. Sequences were processed using the Divisive Amplicon Denoising Algorithm (DADA2; Callahan et al. 2016) implemented in the DADA2 package v1.8 in R v4.0.5, following the approach described by Forster et al. (2019) for hypervariable taxonomic marker genes from metabarcoding studies. The algorithm was applied to V9 SSU rRNA gene sequences with settings optimized for Illumina runs: filterAndTrim was used with truncLen = 80, maxEE = 1, and minOverlap = 20. Afterwards, chimeras were removed with the *uchime‐denovo* algorithm in VSEARCH (Edgar et al. 2011). The remaining sequences were then taxonomically assigned using the PR^2^ database (Guillou et al. 2013) with the LCA (syntax cut‐off of 0.8; Edgar 2016). To reduce bias introduced by PCR and HTS, replicates were pooled. Finally, the ASV table was cleared from singletons, unassigned eukaryotes, Streptophyta, and metazoan sequences to reduce uninformative reads. The resulting ASV‐to‐sample matrix was then used for all downstream statistical analyses (File S4). To determine the saturation levels of the samples and to evaluate the sequencing depth, rarity curves were generated using the rarecurve function in vegan. After removing singletons, unassigned sequences, and non‐target ASVs (Metazoa, Streptophyta), between 111,796 (min) and 1,168,080 (max) high‐quality sequences were obtained (File S5). Rarefaction analyses indicated near‐complete sampling for most samples, after normalization of each sample to the smallest read number (111,796) (File S6).

### 2.6 Statistical Analysis

Alpha diversity metrics, including the Shannon–Wiener Index (H´), Simpson Index (D), ASV richness, and evenness, were analyzed using the rarefied dataset standardized to the smallest sequence size across all samples. We analyzed the effects of light treatment and upwelling intensity on diversity using linear mixed‐effects models implemented in the lme4 package in R. Model parameters and variance components were estimated using restricted maximum likelihood (REML). To assess the significance of fixed effects, we refitted the models using maximum likelihood (ML) and applied Type III Wald *χ* ^2^ tests via the car package. The random effect variance for mesocosm approached zero, which is expected for designs with few groups, but the term was retained to respect the experimental structure.

Beta diversity analysis was conducted using Bray–Curtis dissimilarity on Hellinger‐transformed datasets, which were visualized through non‐metric multidimensional scaling (NMDS; Legendre and Gallagher 2001; Rao 1995). Significant differences in protistan plankton community compositions induced by mesocosm manipulations were assessed using PERMANOVA (9999 permutations) via the adonis2 function. All multivariate analyses (PERMANOVA) were performed with permutations constrained within mesocosms using the strata = Mesokosmos option, ensuring that treatment effects were tested at the correct level of independence. Correlations between physicochemical and biological parameters were integrated into the ordination analysis using the envfit function available in vegan. Trait assignment was conducted by matching taxonomic identities of ASVs to entries in the protist functional trait database (https://www.seanoe.org/data/00405/51662/, accessed on September 22, 2022; Ramond et al. 2018). Functional traits of biotic parameters such as ingestion modes (osmotrophic, saprotrophic, intern, extern) and symbiotic lifestyles (parasitic, commensalistic, mutualistic photosynthesis) were prioritized, whereas traits like shape and structural features were excluded. The morphological traits were excluded because these characteristics are less directly linked to ecological functions such as nutrient acquisition, trophic interactions, or symbiotic relationships.

Taxonomic variations induced by experimental treatments were examined at the division level to provide a descriptive overview of protistan plankton community composition. Relative abundances of the most common groups (cumulative read abundance > 10%) are reported, highlighting patterns across treatments. No formal statistical tests were performed; the results are intended to describe trends and differences in community composition. The data analysis was conducted using RStudio v4.0.5 (R Core Team 2023), employing various statistical and graphical packages, including vegan v2.6–2 (Oksanen et al. 2022), stats v4.3.0 (R Core Team 2021), car v3.0–11 (Fox and Weisberg 2019), relaimpo v2.2–6 (Grömping 2006), and rstatix v0.7.0 (Kassambara 2021). Visual representation of the data was performed using ggplot2 (Wickham 2016) alongside base R functionalities.

## 3 Results

### 3.1 Alpha Diversity of Protistan Plankton Communities in the Humboldt Current System

**FIGURE 2:**
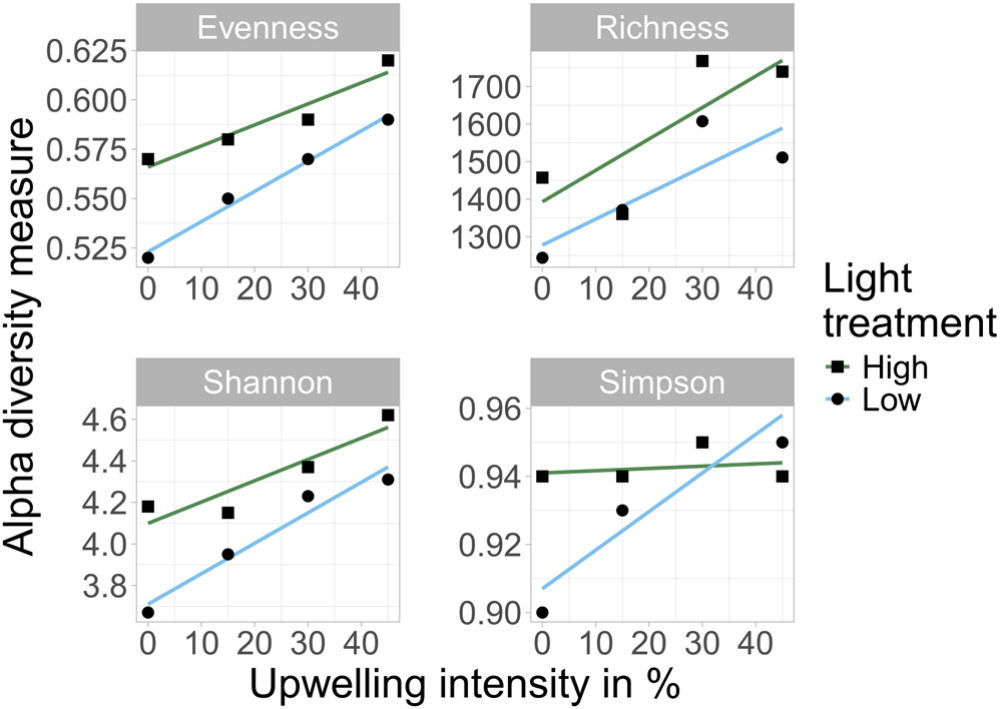
ASV richness, evenness, Shannon Index, and Simpson Index dependent on upwelling intensity and light manipulation derived from integrating all time points (HL 30% hidden behind LL30). The Shannon and Simpson index were calculated for the rarefied data set. The light treatments are colored. Linear trendlines and coefficient of determination (*R* ^2^) for each light treatment were calculated using linear regression analysis.

The alpha diversity metrics, including evenness and ASV richness, alongside the Shannon (H´) and Simpson (D) indices, exhibited greater diversity in HL mesocosms (0.59 ± 0.07, 1581 ± 376, 4.33 ± 0.54, and 0.94 ± 0.05, respectively) compared with LL mesocosms (0.56 ± 0.06, 1433 ± 435, 4.04 ± 0.46, and 0.94 ± 0.03, respectively) (refer to File S7 for raw data). The artificially induced upwelling treatments substantially influenced these diversity metrics across both HL and LL conditions (Figure 2). Evenness demonstrated a positive correlation with upwelling intensity (*R* ^2^ = 0.91 for HL; *R* ^2^ = 0.99 for LL), increasing from 0.57 ± 0.06 (HL0) to 0.62 ± 0.10 (HL45) and from 0.52 ± 0.07 (LL0) to 0.59 ± 0.07 (LL45). A similar trend was observed in the Shannon Index (H´), with lower diversities recorded at 0% upwelling (HL: 4.18 ± 0.47; LL: 3.67 ± 0.53) and higher diversities at 45% upwelling (HL: 4.62 ± 0.84; LL: 4.31 ± 0.47). Although the Simpson Index (D) showed minimal variation, significant deviations were identified under LL treatments in response to DW addition (LL0: 0.90 ± 0.05; LL45: 0.95 ± 0.03). In contrast, D remained relatively stable across HL treatments (*R* ^2^ = 0.07; HL0: 0.94 ± 0.03; HL45: 0.94 ± 0.09).

ASV richness displayed a distinct pattern in both HL and LL mesocosms, where the 30% upwelling intensity yielded the highest ASV richness values (HL30: 1768 ± 279; LL30: 1607 ± 416), surpassing those recorded at 45% upwelling intensity within each respective light treatment.

Despite apparent trends for an increased diversity under HL, linear mixed‐effects models indicated that light had no significant effect on diversity metrics (*p* > 0.29) (Tables 2 and 3). Upwelling intensity had a significant positive effect on richness (*χ* ^2^ = 7.54, *p* = 0.006) and Shannon diversity (*χ* ^2^ = 4.97, *p* = 0.026), indicating increased taxonomic and overall diversity with stronger upwelling. Upwelling effects on evenness were marginal (*χ* ^2^ = 3.59, *p* = 0.058), while Simpson D did not respond significantly (*χ* ^2^ = 0.24, *p* = 0.628). No significant light × upwelling interactions were detected for any metric (all *p* > 0.18). Across all alpha diversity metrics, the fixed‐effect coefficients showed only small differences between light treatments, consistent with the non‐significant effects detected in the ML‐based *χ* ^2^ tests (Tables 2 and 3). Upwelling intensity had consistently positive coefficient estimates for richness, evenness, and Shannon diversity, indicating increasing diversity with stronger DW addition, whereas Simpson D showed only minimal changes in response to upwelling. Interaction terms between light and upwelling were small in magnitude for all metrics.

**TABLE 2:** Type III Wald *χ* ^2^ test results for each alpha diversity measurement (Richness, Evenness, Shannon and Simpson); see File S8 for Shapiro–Wilk and Levene's test results verifying normality and homogeneity of variances.

Alpha diversity	Treatment	*χ* ^2^	*p*‐value
ASV richness	Light treatment	0.155	0.694
	Upwelling intensity	7.535	0.006
	Light × upwelling	1.777	0.183
Evenness	Light treatment	1.102	0.294
	Upwelling intensity	35.887	0.058
	Light × upwelling	0.392	0.531
Shannon Index H´	Light treatment	14.922	0.222
	Upwelling intensity	49.655	0.026
	Light × upwelling	0.346	0.556
Simpson Index D	Light treatment	0.997	0.318
	Upwelling intensity	0.235	0.628
	Light × upwelling	0.249	0.618

**TABLE 3:** Fixed‐effects estimates for each alpha diversity measurement (richness, evenness, Shannon and Simpson).

Alpha diversity	Term	Estimate	SE	df	*t*‐value	*p*‐value
ASV richness	(Intercept)	1406.27	109.44	20.17	12.850	< 0.001
	Light	−56.48	154.77	20.17	−0.365	0.719
	Upwelling	11.93	4.50	42.648	2.653	0.011
	Light:upwelling	−8.46	6.36	42.648	−1.330	0.191
Evenness	(Intercept)	0.61	0.02	14.903	38.251	< 0.001
	Light	−0.02	0.02	14.903	−0.957	0.354
	Upwelling	0.001	0.001	17.795	0.911	0.374
	Light:upwelling	−0.001	0.001	17.795	−1.344	0.196
Shannon Index H´	(Intercept)	450.78	0.15	10.232	29.566	< 0.001
	Light	−0.17	0.22	10.232	−0.783	0.451
	Upwelling	0.006	0.01	21.148	0.727	0.475
	Light:upwelling	−0.015	0.01	21.148	−1.346	0.192
Simpson Index D	(Intercept)	0.95	0.01	35.436	85.638	< 0.001
	Light	−0.01	0.02	35.436	−0.837	0.408
	Upwelling	0.001	0.001	8.654	0.867	0.409
	Light:upwelling	−0.001	0.001	8.654	−0.130	0.899

### 3.2 Dissimilarities Between Mesocosm Specific Protistan Plankton Communities

**FIGURE 3:**
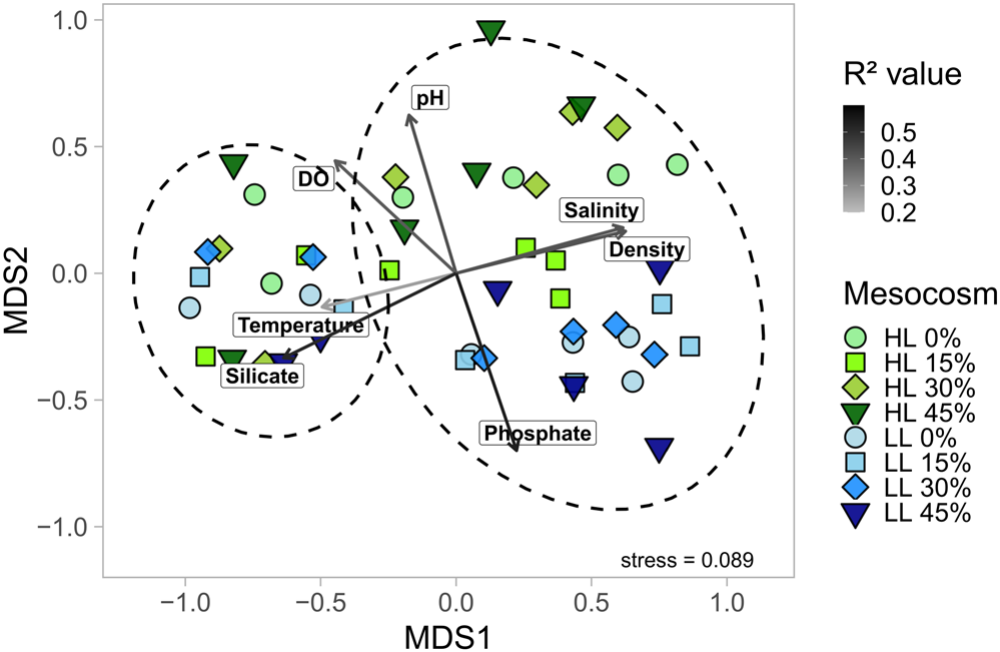
NMDS of protistan plankton community structures based on Bray–Curtis distances. The light treatments are colored and the upwelling intensities are highlighted by symbols. Black circles are the 95% confidence intervals of samples pre‐ and post upwelling. DO, dissolved oxygen.

The NMDS (stress: 0.089) analysis, based on Bray–Curtis dissimilarities, illustrated significant beta diversity variations across samples subjected to different light and upwelling treatments (Figure 3). These variations were evaluated using PERMANOVA (File S9), which revealed that light treatments induced significant differences in protistan plankton communities (PERMANOVA, *F* = 3.95, *R* ^2^ = 0.07, *p* < 0.001). During the pre‐upwelling phase, mesocosm communities differed along one ordination gradient (represented as NMDS axis 1), with HL (high‐light) communities distinctly clustered from LL (low‐light) samples. Post‐upwelling, the HL samples diverged further along a secondary gradient (represented as NMDS axis 2), positioning in the upper range, while LL communities clustered in the lower range. Pre‐upwelling samples remained predominantly at lower NMDS axis 1 values, whereas post‐upwelling samples shifted toward higher axis values. Notably, HL0 and LL0 mesocosms adhered to this pattern despite the absence of nutrient‐rich DW exposure or upwelling treatments. Although the specific upwelling intensities significantly influenced protistan communities (PERMANOVA, *F* = 4.5, *R* ^2^ = 0.09, *p* < 0.001), no apparent pattern was observed in the NMDS.

Environmental factors that significantly correlated with protistan community structures were examined using the envfit function (Figure 3 and File S10). Temperature and silicate concentrations significantly correlated with NMDS axis 1 and were associated with pre‐upwelling samples (*R* ^2^ = 0.26 and 0.52, respectively; *p* < 0.01). Conversely, high salinity and density were linked to post‐upwelling communities (*R* ^2^ = 0.42; *p* < 0.0001). The separation along NMDS axis 2 between HL and LL samples corresponded to phosphate concentrations, dissolved oxygen levels, and pH. Phosphate concentrations were primarily associated with LL samples (*R* ^2^ = 0.53; *p* = 0.0001), whereas higher pH and dissolved oxygen levels correlated with HL communities (*R* ^2^ = 0.40 and 0.42, respectively; *p* < 0.001).

**FIGURE 4:**
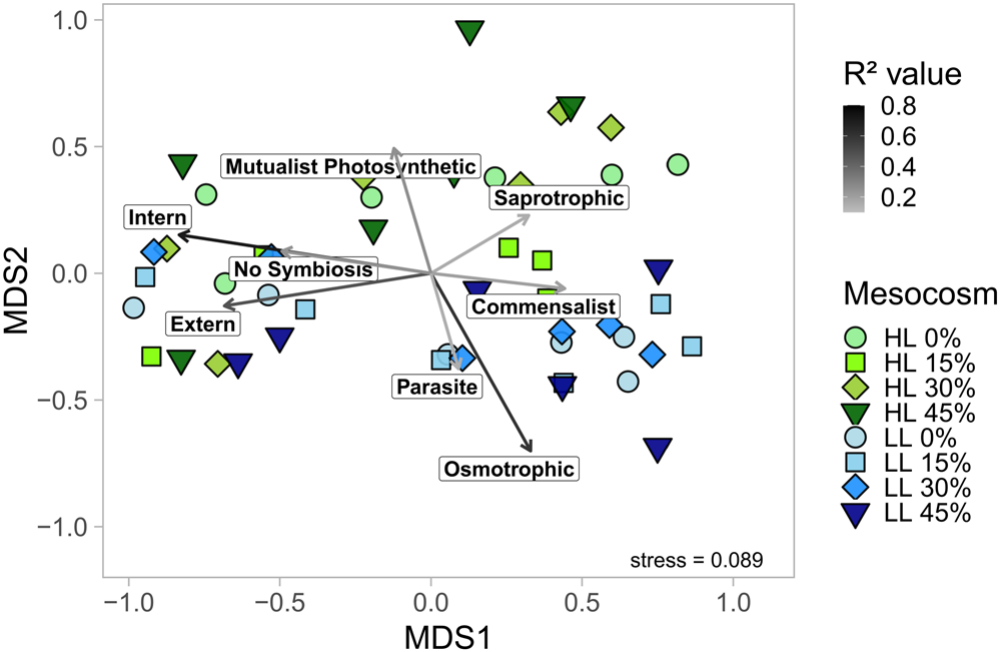
NMDS of protistan plankton community structures based on Bray–Curtis distances with significantly correlated biotic functions. The light treatments are colored and the upwelling intensities are highlighted by symbols. Intern: Internal ingestion; Extern: External ingestion; Mutualist: Mutualistic.

The functional traits of protistan plankton were further analyzed for significant correlations to NMDS structures (Figure 4 and File S10). Annotated symbiosis traits, along with internal and external phagotrophic modes of ingestion, exhibited significant correlations with NMDS axis 1 (*R* ^2^ = 0.25, 0.72, 0.48, respectively; *p* < 0.01). These traits were most prominent in the initial protistan communities prior to DW addition. Saprotrophic and commensalistic relationships correlated with the pre‐to‐post‐upwelling gradient but were more prevalent in post‐DW addition communities (*R* ^2^ = 0.16 and 0.20, respectively; *p* < 0.05). Saprotrophs were primarily associated with HL samples, whereas commensalistic taxa exhibited higher relative abundances in LL mesocosms. Mutualistic photosynthetic relationships were significantly associated with NMDS axis 2 (*R* ^2^ = 0.25; *p* = 0.0013), dominating the HL protistan communities. Parasitic and osmotrophic taxa correlated with LL samples post‐DW addition and were significantly linked to NMDS axis 2 (*R* ^2^ = 0.15 and 0.60, respectively; *p* < 0.05).

### 3.3 Taxonomic Composition of Protistan Plankton Communities

**FIGURE 5:**
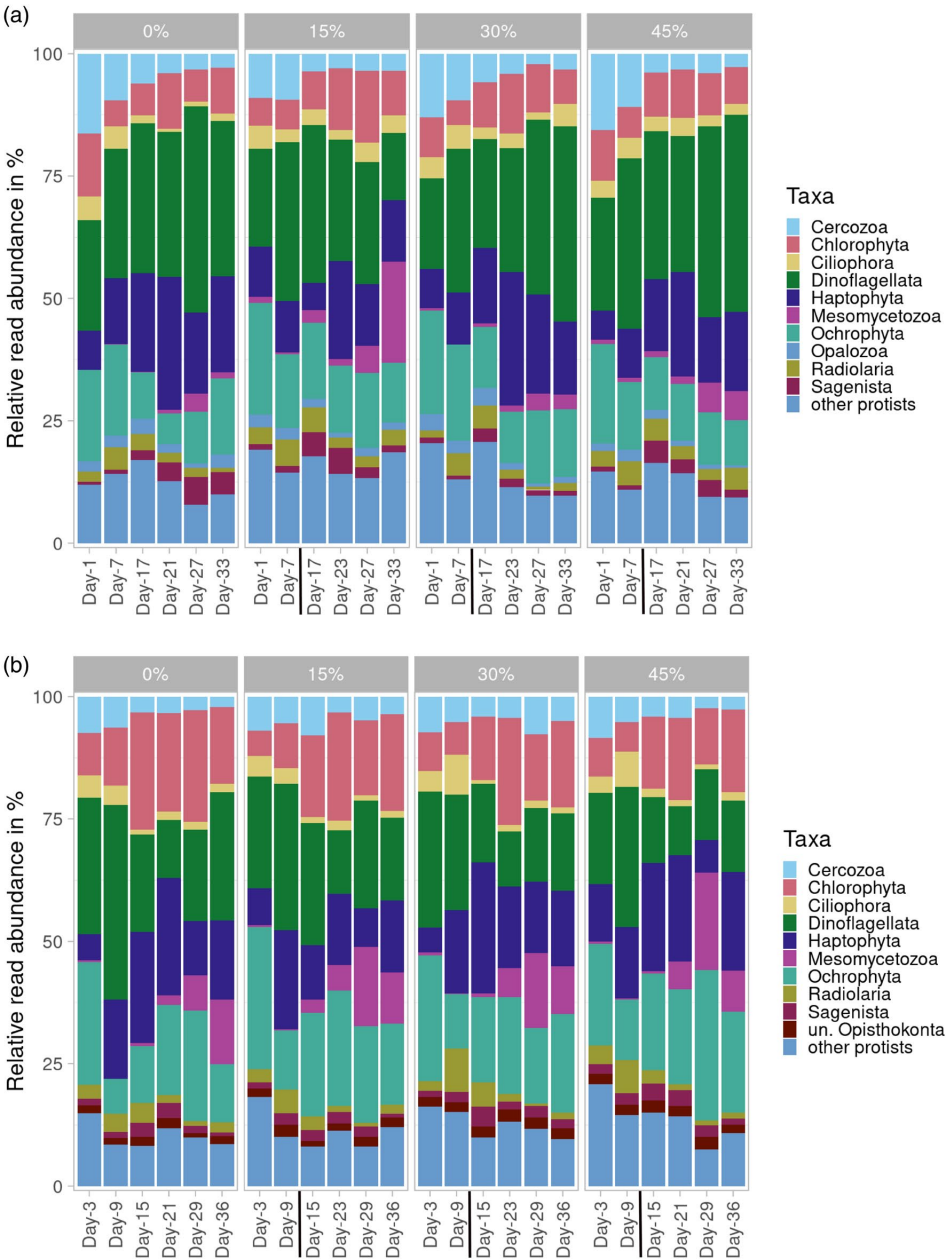
Taxonomic inventories of protistan plankton ASVs reads for (a) HL mesocosm and (b) LL mesocosms. The bars represent the relative abundance of ASVs assigned to each of the different taxon groups at the division level. Black line indicates deep‐water addition. We further provide a line plot for the top 5 divisions across key treatments to illustrate temporal trajectories more clearly (File S11).

The dominant division observed across all samples was Dinoflagellata (Supergroup TSAR—Alveolata), representing 24.6% ± 8.5% of the relative abundance of sequence reads. Ochrophyta (Supergroup TSAR—Stramenopiles) followed as the second most abundant group (16.6% ± 5.5%), succeeded by Haptophyta (Supergroup Haptista) (15.0% ± 5.9%) and Chlorophyta (Supergroup Archaeplastida) (11.6% ± 5.2%). Less prevalent phyla included Cercozoa (Supergroup TSAR—Rhizaria), Mesomycetozoa (Supergroup Ophistokonta, mostly represented by Abeoformidae‐Group MAIP 2 (MAIP: Marine Ichthyosporea)), Radiolaria (Supergroup TSAR—Rhizaria), and Ciliophora (Supergroup TSAR—Alveolata), each contributing proportions below 10% but exceeding 1%. Notably, the succession and relative proportions of read abundances varied according to the light treatments (Figure 5, Table 4).

**TABLE 4:** Dominant protistan plankton groups associated to the specific treatments: Chlorophyta, Dinoflagellata, Haptophyta, and diatoms arranged in decreasing order of relative abundance for the specific treatment.

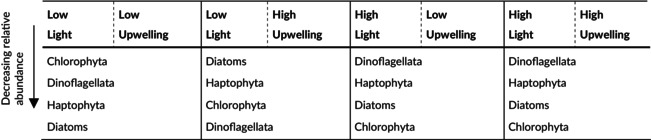

In the high‐light (HL) mesocosms, Dinoflagellata constituted 29.1% ± 7.3% of the reads, contrasting with 20.1% ± 7.3% observed in the low‐light (LL) mesocosms. In LL treatments, Ochrophyta (18.8% ± 5.9%), Haptophyta (15.2% ± 6.1%), and Chlorophyta (14.4% ± 5.7%) followed a sequential pattern of dominance based on read abundance. Conversely, in HL mesocosms, Haptophyta (14.8% ± 6.0%) emerged as the second most abundant, followed by Ochrophyta (14.3% ± 4.2%) and Chlorophyta (8.8% ± 2.6%). The relative abundance of Haptophyta exhibited minimal variation between light treatments; however, Chlorophyta reads were nearly halved in HL compared with LL samples.

Among the less dominant taxa, Cercozoa (6.3% ± 4.3%) were more prevalent in HL mesocosms, followed by Ciliophora (3.1% ± 1.3%) and Radiolaria (3.0% ± 1.4%), with Mesomycetozoa contributing 2.6% ± 4.2%. In LL samples, Mesomycetozoa accounted for more than double their relative proportion observed in HL communities (5.3% ± 6.0%), while Cercozoa (4.9% ± 1.9%), Radiolaria (2.7% ± 2.0%), and Ciliophora (2.6% ± 2.0%) were present at lower proportions. The residual frequency of reads comprised approximately 18% and 16% across HL and LL samples, respectively, distributed among 24 additional taxonomic groups (File S2).

## 4 Discussion

### 4.1 Effect of Light and Upwelling Scenarios on Regional Protistan Plankton Diversity

Future implications of anthropogenic climate change suggest significant alterations in coastal light regimes and upwelling dynamics, substantially influencing the EBUS off Peru. Changes in atmospheric circulation, coastal wind patterns, and ocean stratification are predicted to modify the timing, intensity, and duration of upwelling events, potentially reducing nutrient delivery to the euphotic zone (Bakun et al. 2015; Messié and Chavez 2015). Concurrently, variations in cloud cover, precipitation, and increased water turbidity are expected to alter the light environment, influencing photosynthetic activity and primary production (Doney et al. 2012; Seitzinger et al. 2010). Understanding protistan plankton responses to these environmental changes is therefore crucial. The investigation of varying light intensities combined with multiple upwelling scenarios revealed significant impacts on protistan diversity, community composition, and trophic interactions as hypothesized. Because the upwelling manipulation consisted of a single DW pulse, part of the initial increase in diversity observed in the mesocosms may reflect the mixing of surface and DW microbial communities. Without sequencing the DW, we cannot determine the exact contribution of dispersal. However, selective responses among taxonomic and functional groups, rather than uniform increases across all taxa, indicate that nutrient‐induced growth dynamics played a substantial role in shaping post‐pulse community trajectories. The findings presented in this study provide valuable insights into potential future shifts in unicellular eukaryotic dynamics within EBUS under climate change.

### 4.2 Immigrant Plankton Types Enhance Protistan Plankton Diversity by Simulated Upwelling

The simulated upwelling scenarios demonstrated a notable increase in alpha diversity, reflecting both the introduction of DW taxa and community restructuring under altered nutrient and light regimes. This observation partly contradicts the paradox of enrichment, which posits that increased resource availability promotes dominance by fast‐growing opportunists and thus reduces overall diversity (Grover 1990; Papanikolopoulou et al. 2018; Tubay et al. 2013). While some of the observed diversity increase may be attributed to the effect from DW microbial assemblages, the sustained shifts in community composition across treatments suggest that ecological interactions following nutrient enrichment also contributed to the observed patterns. The absence of a pronounced plankton bloom‐induced reduction in diversity may be attributable to the experimental upwelling mode. As opposed to continuous upwelling observed in natural systems, the single‐pulse upwelling applied in this study likely mitigated the extinction pressures on less adapted species (Ortiz et al. 2022; Papanikolopoulou et al. 2018). These findings align with microcosm experiments examining diversity responses to nutrient supply (Anderson et al. 2022; Dittrich et al. 2023). Dittrich et al. (2023) identified a positive correlation between species richness and nutrient concentrations, suggesting that such treatments do not necessarily eliminate less adapted species. Similarly, Anderson et al. (2022) reported that incubations with limited nutrient concentrations exhibited the lowest evenness, consistent with the results observed in this study.

In contrast to the paradox of enrichment, the inevitable nutrient depletion within the mesocosms may favor the dominance of taxa acclimated to nutrient scarcity, thereby reducing alpha diversity (Grover 1990; Margalef 1978; Papanikolopoulou et al. 2018). This phenomenon is further elucidated by the absence of immigrant plankton types within the DW addition in the 0% upwelling mesocosms, preventing an increase in diversity due to the merger of local and immigrant plankton communities. This observation aligns with the ‘dispersal‐driven diversity theory’ (Cadotte 2006; Clayton et al. 2013). Consequently, the results highlight the role of immigrant plankton in enhancing diversity within mesocosms subjected to 15%–45% upwelling simulations. The findings are consistent with recent field studies conducted in the California Current System, which emphasized that moderate, intermittent upwelling conditions promote the highest diversity (Harvey et al. 2021). Nevertheless, defining intermediate disturbance in the context of upwelling intensity requires further investigation, given the challenges associated with quantifying the implications across the entire disturbance spectrum (Huston 1979).

### 4.3 Fluctuations of Phytoplankton Diversity Induced by Distinct Light Intensities

The variations induced by distinct light treatments revealed alterations in alpha diversity, which can profoundly impact ecosystem functioning (Brose and Hillebrand 2016). Changes in local diversity can influence primary production and nutrient cycling, alter trophic and competitive interactions, and affect the capacity of communities to maintain functional stability under environmental fluctuations. Moreover, shifts in local (alpha) diversity may cascade to larger spatial scales by modifying species turnover (beta diversity), potentially reducing the resilience and functional redundancy of the broader metacommunity. The experimental simulation of natural austral summer conditions, characterized by high‐light (HL) intensity, exhibited generally higher alpha diversity compared with the austral winter simulation under low‐light (LL) intensity. These findings align with previous studies on prokaryotes, phytoplankton, and zooplankton communities (Gong et al. 2023; Suleiman et al. 2022; Wang et al. 2023). However, contrary results have also been reported, demonstrating a negative correlation between phytoplankton diversity and light intensity (Flöder et al. 2002; Rodrigues et al. 2015).

This apparent discrepancy may stem from species‐specific physiological responses to varying light intensities, which dictate optimal photosynthetic activity thresholds (Huisman et al. 1999; Yang et al. 2020). Since the mid‐1970s, light intensity has been theorized as a pivotal factor influencing niche separation, thereby structuring coexistence and exclusion within plankton communities (Flöder et al. 2002; Litchman 1998; van Gemerden 1974). Huisman et al. (1999) confirmed theoretical predictions regarding species‐specific “critical light intensities,” defined as the minimum light availability required for steady‐state abundance. Under light limitation, species with the lowest critical light intensity thresholds dominate the community, leading to reduced alpha diversity.

These results suggest that competition for light resembles exclusion principles observed in nutrient dynamics (Flöder et al. 2002; Hardin 1960; Huisman et al. 1999). The findings further indicate that the resident plankton communities along the Peruvian coast are highly adapted to natural light regimes and are subject to immediate limitations under reduced light conditions. Nonetheless, certain plankton species appear particularly well suited to maintain functional activity under low‐light conditions.

### 4.4 Effect of Distinct Upwelling Intensities on Protistan Plankton Structure in a Mimicked Austral Winter Situation

During the initial phase of the austral winter simulation, diatom relative abundance declined, which correlates with elevated silicic acid concentrations, as diatoms utilize this compound for constructing silica‐based frustules (Lopez et al. 2005). Silica assimilation by diatoms is light‐dependent (Blank and Sullivan 1979), and under reduced light intensities, this uptake is diminished, which explains the elevated silicic acid levels observed in the austral winter mesocosms compared with those of the austral summer simulation (Azam and Chisholm 1976; Taylor 1985). The decline in diatom abundance was accompanied by a corresponding reduction in dissolved oxygen, indicating a collapse in photosynthetic activity due to the rapid light reduction implemented to simulate austral winter conditions (Alderkamp et al. 2019; Sekerci and Petrovskii 2018).

The injection of DW introduced nutrient concentrations that mirrored those found in natural upwelling systems, albeit temporarily (Hauschildt et al. 2021; Igarza et al. 2019; Messié et al. 2009). This nutrient input instigated permanent changes in community composition, facilitating a reversal in the initial decline of diatom proportions. Post‐DW addition, diatoms dominated, reflecting their strong nutrient‐driven competitive advantage. Diatoms along the Peruvian coast exhibit rapid responses to nutrient enrichment, often outcompeting other species, consistent with their fast nutrient uptake and growth rates (Brown et al. 2008; Landry et al. 2000; Van Dam 1982). The sharp decline in silicic acid concentration, likely attributable to diatom biomineralization, supports this observation. Prominent groups included Polar‐centric‐Mediophyceae (e.g., *Thalassiosira* spp., *Minidiscus* spp., and *Skeletonema* spp.), which are ubiquitous primary producers within the HCS (González et al. 2007; Montero et al. 2007). Adaptation to low‐light conditions by these diatoms is widely observed in marine environments (Geider et al. 1996; Masuda et al. 2021; Talmy et al. 2013). Moreover, diatoms are significant contributors to the biological carbon pump within the HCS, particularly during austral winter (González et al. 2009; Messié and Chavez 2015).

Under nutrient‐deficient conditions, Chlorophyta emerged as prevalent contributors, primarily exhibiting osmotrophic foraging strategies. This aligns well with our findings, as osmotrophs—generally smaller organisms—show a competitive advantage under nutrient‐deficient environments, whereas nutrient‐rich conditions favor larger cells (Våge et al. 2013). This divergence in size‐class distribution, induced by distinct upwelling scenarios, has profound implications for food web structure and ecosystem services (Stibor et al. 2019). Although Chlorophyta are typically less abundant in these waters, their occurrence is notably higher during El Niño events within the HCS due to nutrient‐deficient conditions (Arntz et al. 2006; DiTullio et al. 2005; Mogollón and Calil 2017). Previous studies have also identified Chlorophyta in mesocosm experiments, suggesting that turbulent waters suppress Chlorophyta due to niche segregation, despite the mesocosms' ability to transmit partial turbulence from the ocean (Bach et al. 2020; Margalef 1978; Sharma et al. 2021). Their strong photoacclimation abilities support efficient photosynthetic activity under low‐light conditions, explaining their elevated abundance under LL and nutrient‐deprived scenarios (Fisher et al. 1989; Masojídek et al. 1999; Parsons et al. 2013).

Abeoformidae‐Group‐MAIP‐2 accounted for the majority of annotated Mesomycetozoa and represented one of the predominant parasitic taxon groups in the austral winter simulation. Although ecological knowledge and parasite–host dynamics of Ichthyosporea remain limited, documented hosts include diatoms and fish (Käse et al. 2021; Marshall and Berbee 2011; Rowley et al. 2013). The results from this study corroborate these findings, as diatom proportions and the introduction of fish in the mesocosms coincided with the relative abundance and presence of Ichthyosporea. While this correlation is consistent with previously reported host associations, it should be interpreted as a plausible hypothesis rather than evidence of direct parasitic interactions, as no data on fish health, infection status, or diatom–parasite interactions were collected. Additionally, Abeoformidae (Group MAIP) is linked to enhanced carbon flux within global oceans, signifying its potential role in the biological carbon pump (Guidi et al. 2016).

The introduction of fish larvae represents an important ecological manipulation that likely influenced the subsequent dynamics of the protistan plankton community. By preying on zooplankton, the larvae may have induced a trophic cascade that reduced grazing pressure on protistan plankton, thereby indirectly promoting the growth of smaller photoautotrophic groups. In addition, nutrient excretion by the fish larvae could have locally enriched dissolved nutrient pools, stimulating primary production and favoring fast‐growing taxa such as Chlorophyta (Chen et al. 2021). These processes together may have contributed to the shifts observed in community composition during the latter phase of the experiment, including the proliferation of parasitic groups such as Dinoflagellata and Mesomycetozoa. The presence of fish hosts might also have provided additional substrates or hosts for these parasitic taxa, compounding the complexity of the observed interactions. Importantly, the introduction of fish larvae constitutes a potential confounding factor when interpreting the effects of light and upwelling treatments, particularly for data collected after day 17. The trophic and nutrient‐mediated effects induced by the larvae could have interacted with the experimental treatments in non‐additive ways. For example, enhanced nutrient regeneration under low‐light or upwelling conditions might have amplified treatment‐specific responses, whereas changes in grazer abundance could have modified the apparent effects of light intensity on protistan structure and productivity. Consequently, the responses observed after day 17 should be interpreted as reflecting combined influences of the physical (light and upwelling) and biological (fish‐mediated) perturbations, rather than as isolated effects of the primary treatments. Future studies could disentangle these factors by maintaining parallel mesocosms without fish larvae or by explicitly incorporating higher‐trophic‐level manipulations into the experimental design.

### 4.5 Effect of Distinct Upwelling Intensities on Protistan Plankton Structure During Austral Summer

Dinoflagellates dominated relative sequence reads during the austral summer simulation, aligning with studies conducted in the HCS, where dinoflagellates consistently prevailed within the protistan plankton community (Min et al. 2023). Their substantial contribution to carbon flux into deeper oceanic layers during austral summer has been well documented (González et al. 2009). The elevated photosynthetic activity of dinoflagellates under high‐light conditions relative to diatoms and green algae resulted in a notable reduction of approximately 50% in the proportions of chlorophytes and diatoms compared with the austral winter simulation (Parsons et al. 2013). This photosynthetic advantage provided dinoflagellates with a substantial competitive edge (Huisman et al. 1999; Parsons et al. 2013).

While diatoms have been reported to dominate the Peruvian HCS during austral summer (Min et al. 2023), the intensified stratification and reduced turbulence in the mesocosms deviated from optimal conditions for diatom proliferation. Consequently, dinoflagellates maintained a competitive advantage even under nutrient‐enriched scenarios (Margalef 1978). The capacity of dinoflagellates to acquire nutrients through vertical migration likely further enhanced their success (Heaney and Eppley 1981; Peacock and Kudela 2014). The consistent prevalence of dinoflagellates across all upwelling treatments suggested that nutrients served as a secondary selective factor. With their diverse ecological strategies—including parasitism, symbiosis, heterotrophy, mixotrophy, and phototrophy—dinoflagellates exhibited a remarkable adaptability to varying environmental conditions (Hansen 2011; Santi et al. 2019; Stoecker 1999). Nevertheless, internal shifts were evident, as many dinoflagellate species remain reliant on dissolved nutrient availability (Wu et al. 2020).

The increase in amplicon sequence variants (ASVs) assigned to Syndiniales (Dino Group‐I‐Clade‐4) reflected a shift within dinoflagellates from heterotrophic or mixotrophic modes of nutrition toward parasitism under nutrient‐deficient conditions, underscoring their functional diversity (Santi et al. 2019). This trophic shift was further corroborated by Non‐Metric Multidimensional Scaling (NMDS) analysis, suggesting potential implications for trophic transfer efficiency (Ward and Follows 2016). Syndiniales, an exclusively parasitic taxon, are known to infect a wide range of hosts (dinoflagellates, acantharians, diatoms, radiolarians, Cercozoa, and metazoans) (Guillou et al. 2008; Käse et al. 2021; Min et al. 2023; Nagarkar and Palenik 2023; Siano et al. 2011). Their role in top‐down control of protistan and zooplankton communities, coupled with their contribution to particulate carbon flux, positions them as key players in oceanic carbon cycling (Anderson et al. 2023).

Evidence of competition within Dinophyceae was provided by the differential relative abundances of specific families, such as Suessiaceae and Prorocentraceae. Suessiaceae, in particular, exhibit diverse ecological strategies, including free‐living, symbiotic, and parasitic forms (Hehenberger et al. 2018; Montresor et al. 1999; Siano et al. 2010). The correlated abundance patterns of Suessiaceae and Syndiniales suggest that many Suessiaceae may occupy parasitic niches or serve as hosts for Syndiniales. However, confirmed parasite–host relationships between these groups remain absent in the literature (Guillou et al. 2008; Nagarkar and Palenik 2023).

Haptophyta, primarily represented by Phaeocystaceae, also emerged as a dominant group across mesocosms during both austral summer and winter simulations. This group plays a significant role in oceanic carbon cycling and potentially contributes to climate regulation through the production of dimethyl sulfide, a precursor of cloud condensation nuclei (Charlson et al. 1987; Schoemann et al. 2005; Smith Jr et al. 1991; Stefels et al. 1995). Their relatively uniform distribution across treatments suggests a robust capacity for photoadaptation within the HCS, consistent with a broad spectrum of light intensities reported for *Phaeocystis* spp. (Peperzak et al. 2000; Schoemann et al. 2005; van Hilst and Smith 2002). While *Phaeocystis* species thrive in nutrient‐rich waters, no significant association with higher upwelling intensities was observed in the present study (Schoemann et al. 2005; Smith Jr et al. 1991). Their highest relative abundance under nutrient‐depleted conditions indicates a competitive disadvantage relative to the local diatom and dinoflagellate communities under intensified upwelling.

These shifts in community composition and size‐class distribution had cascading effects on trophic interactions and carbon cycling dynamics. Favoring smaller cells and reducing the prevalence of mixotrophic organisms under conditions of reduced upwelling could extend food web length and negatively impact fisheries (Mehner et al. 2018; Stibor et al. 2019; Ward and Follows 2016). Nutrient deficiency was linked to increased parasitism, which intensified top‐down control of phytoplankton, potentially reducing carbon fixation rates (Frenken et al. 2016; Klawonn et al. 2023; Worden et al. 2015). Yet, parasitism has been associated with enhanced particulate carbon fluxes, highlighting the need to quantify these opposing mechanisms (Anderson et al. 2023). In the context of projected equatorward reductions in upwelling and intensifying El Niño–Southern Oscillation events, the results provide valuable insights into future protistan plankton dynamics and their impact on the HCS (Rykaczewski et al. 2015; Wang et al. 2017).

### 4.6 Experimental Considerations

A potential limitation of our study is the relatively small number of mesocosms per treatment, which may constrain the statistical power in detecting subtle treatment effects and interactions. Each light and upwelling treatment was represented by only four replicates—a common constraint in field‐based mesocosm experiments (Czerny et al. 2013; Bach et al. 2020) that nonetheless can limit the ability to fully capture the inherent variability of protistan plankton communities. Previous work emphasized that increased replication is essential to distinguish treatment‐induced effects from natural variability and to allow the application of more robust statistical models (Underwood 1997; Quinn and Keough 2002). Mixed‐effects models, which accommodate temporal and spatial autocorrelation, often require larger sample sizes to produce reliable estimates (Zuur et al. 2009). Although our findings are consistent with comparable upwelling studies (Riebesell et al. 2013; Dittrich et al. 2023), additional replicates in future experiments would enhance the resolution and confidence of detected ecological responses. Addressing this constraint in subsequent studies will be crucial for accurately quantifying the balance between treatment effects and natural variability inherent in these dynamic marine systems.

Although mesocosms offer a powerful tool to isolate and test specific environmental drivers under controlled conditions, they inherently simplify the natural complexity of upwelling and light regimes. In our study, nutrient enrichment was achieved by a single DW injection that, while effective at eliciting community responses, does not fully capture the continuous or multi‐scale variability observed in natural upwelling systems (Bakun et al. 2015; Messié and Chavez 2015). Natural upwelling events are often characterized by a heterogeneous mix of nutrient pulses, variable turbulence, and spatial gradients that influence plankton dispersal and competition (Riebesell et al. 2013; Gruber et al. 2011). A single pulse provides only short‐term enrichment; as nutrients decline, competitive exclusion weakens, and multiple taxa can coexist during early post‐disturbance phases. This dynamic likely contributed to the elevated alpha diversity in high upwelling treatments, deviating from expectations under the classical paradox of enrichment, which assumes persistent nutrient loading. Thus, the diversity peak in our experiment should be interpreted as a short‐term response to abrupt enrichment, rather than a long‐term outcome that would necessarily arise in a system experiencing continual or repeated upwelling. Moreover, in natural systems, recurrent upwelling events continually reset or alter successional trajectories, whereas our design allowed the community to progress uninterrupted after a single disturbance. The constrained mixing and fixed geometric configuration within mesocosms may therefore modulate nutrient uptake rates and trophic interactions differently compared with open ocean conditions (Czerny et al. 2013; Dittrich et al. 2023). These constraints underscore that our findings, while robust under controlled manipulations, should complement in situ observations that capture full natural variability. Future studies integrating high‐resolution field monitoring with mesocosm experiments will be essential to validate and refine our understanding of how variable upwelling intensity and light conditions drive protistan plankton dynamics in the HCS. Especially incorporating water temperature as an additional manipulated factor in such experiments would further clarify how multiple environmental drivers interact to shape community composition, trophic strategies, and ecosystem functioning.

## 5 Conclusion

This study demonstrates that variations in light intensity and upwelling critically shape the diversity and community composition of protistan plankton in EBUS. By simulating distinct upwelling pulses and seasonal light environments, our results reveal that both physical factors act as key regulators, influencing nutrient dynamics, trophic interactions, and ultimately community structure. The observed increase in alpha diversity under upwelling emphasizes the role of immigrant plankton in promoting local diversity, challenging the classical paradox of enrichment and underscoring the importance of dispersal‐driven diversity in these dynamic systems.

Protistan communities respond in complex and sometimes counterintuitive ways to abrupt environmental changes. Short‐term nutrient pulses favored coexistence across taxa rather than competitive exclusion, while species‐specific light adaptations in diatoms, chlorophytes, and dinoflagellates drove niche differentiation which could affect ecosystem productivity. The transition toward smaller, more diverse protistan assemblages may enhance resistance to perturbations by promoting functional redundancy and rapid metabolic turnover. In contrast, the increase of parasitic taxa such as Ichthyosporea suggests potential vulnerabilities in food web stability, as increased host–parasite interactions could dampen recovery after disturbance events. These dual trends indicate that resilience in the HCS may depend on a delicate balance between community plasticity and trophic connectivity. Systems dominated by small, opportunistic taxa may recover rapidly from short‐term fluctuations but remain sensitive to chronic stressors like sustained warming or nutrient imbalance.

This research underscores the value of integrating experimental mesocosm approaches with ecological theory to elucidate the mechanisms governing microbial community dynamics. By linking controlled experimental simulations with theoretical models such as the intermediate disturbance hypothesis and dispersal‐driven diversity theory, our study provides a robust framework for forecasting ecological responses under future climate scenarios. These findings advance the understanding of protistan plankton ecology in upwelling systems and provide practical insights for guiding research and management strategies in a rapidly changing marine environment.

## Author Contributions

**Sven Katzenmeier:** conceptualization (equal), data curation (equal), formal analysis (equal), visualization (equal), writing – original draft (equal), writing – review and editing (equal). **Megan Gross:** data curation (equal), investigation (equal), writing – review and editing (equal). **Thorsten Stoeck:** conceptualization (equal), funding acquisition (equal), methodology (equal), project administration (equal), resources (equal), supervision (equal), validation (equal), writing – review and editing (equal).

## Funding

This study is embedded in the BMBF (Bundesministerium für Bildung und Forschung)‐funded project CUSCO—Coastal Upwelling System in a Changing Ocean (funding label 03F0813A) and financially supported by AQUACOSM (Project BacPro).

## Conflicts of Interest

The authors declare no conflicts of interest.

## Supporting information


**File S1:** ece372827‐sup‐0001‐FileS1.pdf.

**File S2:** ece372827‐sup‐0002‐FileS2.pdf.

**File S3:** ece372827‐sup‐0003‐FileS3.pdf.

**File S4:** ece372827‐sup‐0004‐FileS4.pdf.

**File S5:** ece372827‐sup‐0005‐FileS5.pdf.

## Data Availability

The sequence data files are deposited at the Sequence Read Archive of the National Center for Biotechnology Information under project number PRJNA1286811.
