# Supplementary material for: Protistan Plankton Responses to Variable Light and Upwelling in the Peruvian Humboldt Current System: Insights Into Community Dynamics Under Environmental Change
Source: Ecol Evol. 2026 Jan 12;16(1):e72827. doi: 10.1002/ece3.72827 (PMC12796512; doi:10.1002/ece3.72827)

Supplementary file 1. Mesocosm volume determined through salt addition with uncertainty and nutrient concentrations of the mesocosms. Nutrient concentrations of each KOSMOS after deep-water (DW) addition on day 15 and the 100% DW. It should be noted that the nutrient concentrations do not reflect the concentration directly after DW injection due to the time delay in the process and water sampling. HL: high light; LL: low light; M: mesocosm; OA: Orthosilicic acid; V: Volume.

**KOSMOS**	**V [L]**	**Uncertainty [L]**	**Light**	**Upwelling [%]**	**Nitrat [µmol/L]**	**Phosphat [µmol/L]**	**OA [µmol/L]**
M1	36.440	2.064	HL	0	0.00	0.87	7.90
M2	35.195	2.167	LL	45	11.3	1.83	15.40
M3	35.984	2.242	HL	30	11.1	1.48	12.60
M4	30.743	2.166	LL	30	8.10	1.48	14.10
M5	32.786	454	HL	45	13.30	1.69	12.90
M6	34.116	963	LL	15	3.60	1.22	14.50
M7	33.242	1.507	HL	15	4.60	1.17	11.10
M8	35.097	1.713	LL	0	11.30	1.83	15.40
DW	-	-	-	100	30.60	2.45	15.90

Physico chemical dynamics

Chemical and physical parameters of the mesocosms displayed notable variations throughout the experiment and between treatment conditions (Supplementary file 3 and 4). The pH (Supplementary file 4a) was influenced by the light treatment, consistently higher in the HL mesocosms (pH = 8.2 ± 0.1) compared to the LL mesocosms (pH = 8.0 ± 0.1). The effect of deep-water (DW) treatment on pH was observed on sampling days (LL: day 15; HL: day 17) immediately following its application. The pH declined in proportion to the simulated upwelling intensity and subsequently increased steadily until the conclusion of the experiment. Dissolved oxygen concentrations (Supplementary file 4b) followed a similar trend, being primarily impacted by the light treatment (HL: O_2 = 6.3 ± 0.6 mg/L; LL: O_2 = 5.5 ± 0.4 mg/L), particularly during the initial 20 days. On days 15 (LL) and 17 (HL), following DW addition, the pattern was inverted compared to pH. Higher upwelling intensities generally corresponded to elevated oxygen concentrations (with the exception of HL30 and HL45). Oxygen levels consistently declined under HL treatments but either increased slightly or remained constant in LL mesocosms. Temperature (Supplementary file 4c) exhibited minimal

variation and remained stable (17.9 ± 0.3 °C) without significant differences between light treatments (HL: 17.9 ± 0.4 °C; LL: 17.9 ± 0.3 °C).

Nutrient dynamics were strongly influenced by the artificial upwelling intensities and the indirect effects of light on the biological activity. Concentrations of nitrate, silicate, and phosphate were generally higher in LL-treated mesocosms, although all exhibited marked changes over the course of the experiment. Nitrate (Supplementary file 4d) was depleted prior to DW addition and increased proportionally to the intensities of the upwelling treatments. After reaching a peak, nitrate levels steadily declined. Notably, in the HL0 and LL0 conditions, nitrate concentrations exhibited a slight increase until approximately day 20 before decreasing to marginal levels. Phosphate (Supplementary file 4e) and silicate (Supplementary file 4f) were not depleted prior to DW application. Phosphate concentrations decreased significantly up to days 7/9, while silicate levels remained stable during the pre-DW phase. In the post-DW period, silicate concentrations declined continuously, particularly in the HL45 and LL45 treatments, with a pronounced drop between days 13/15 and 21. Phosphate concentrations peaked immediately following the application of 30% and 45% upwelling intensities and subsequently decreased. Interestingly, phosphate levels in the 0% and 15% upwelling mesocosms increased until day 29 (LL and HL).

Supplementary file 2. Physicochemical and nutrient data for each sample. Supplied as pdf.

Supplementary file 3. pH (a), dissolved oxygen (b), temperature (c), nitrate (d), silicate (e) and phosphate (f) development in the mesocosms. Deep-water addition is highlighted by the red dashed line. Light treatment is colored in green for high light (HL) and blue for low light (LL).

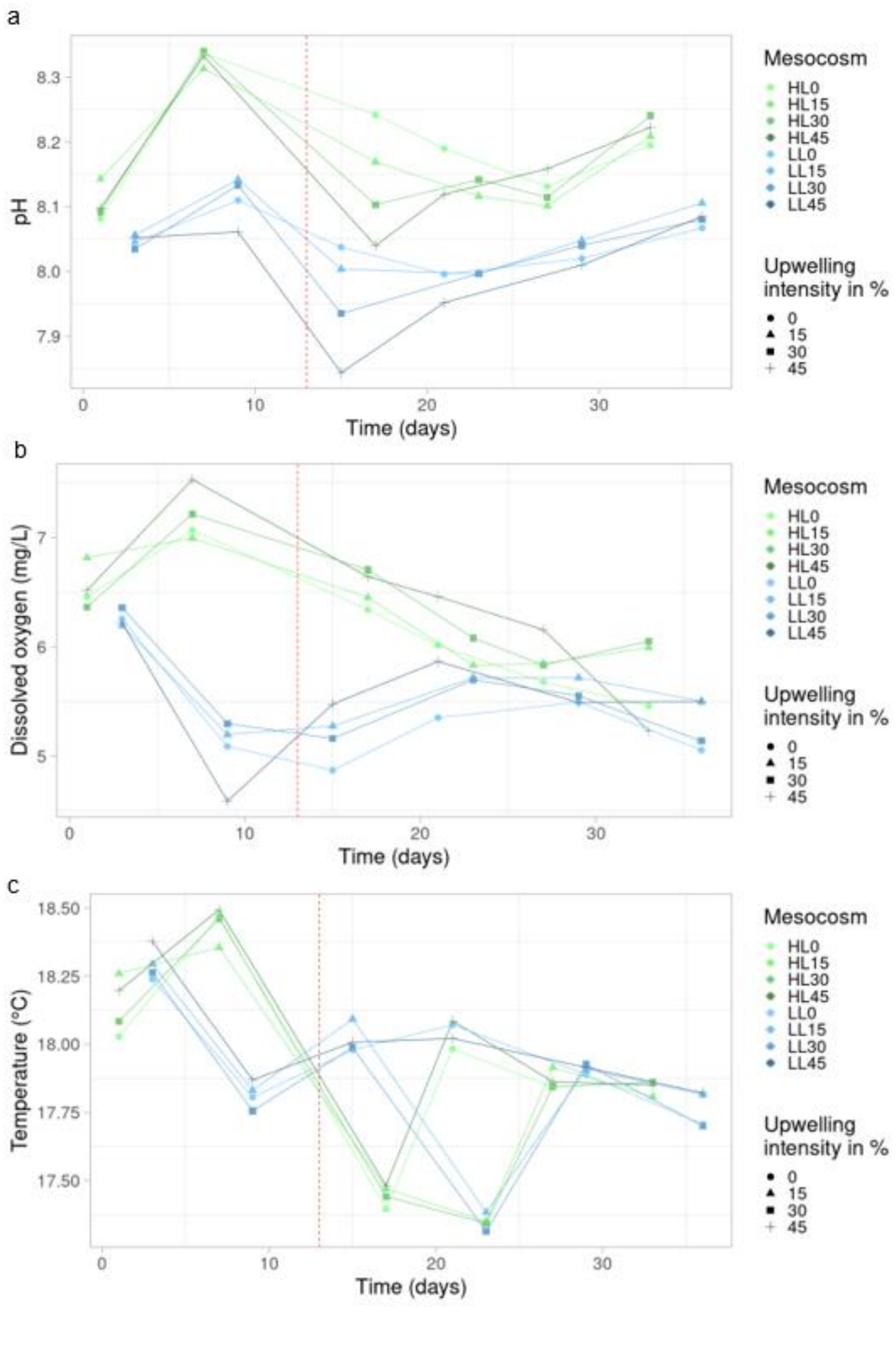

a
pH
8.3
8.2
8.1
8.0
7.9
0
10
20
30
Time (days)
Mesocosm
HL0
HL15
HL30
HL45
LL0
LL15
LL30
LL45
Upwelling intensity in %
0
15
30
45
b
Dissolved oxygen (mg/L)
7
6
5
0
10
20
30
Time (days)
Mesocosm
HL0
HL15
HL30
HL45
LL0
LL15
LL30
LL45
Upwelling intensity in %
0
15
30
45
c
Temperature (°C)
18.50
18.25
18.00
17.75
17.50
0
10
20
30
Time (days)
Mesocosm
HL0
HL15
HL30
HL45
LL0
LL15
LL30
LL45
Upwelling intensity in %
0
15
30
45

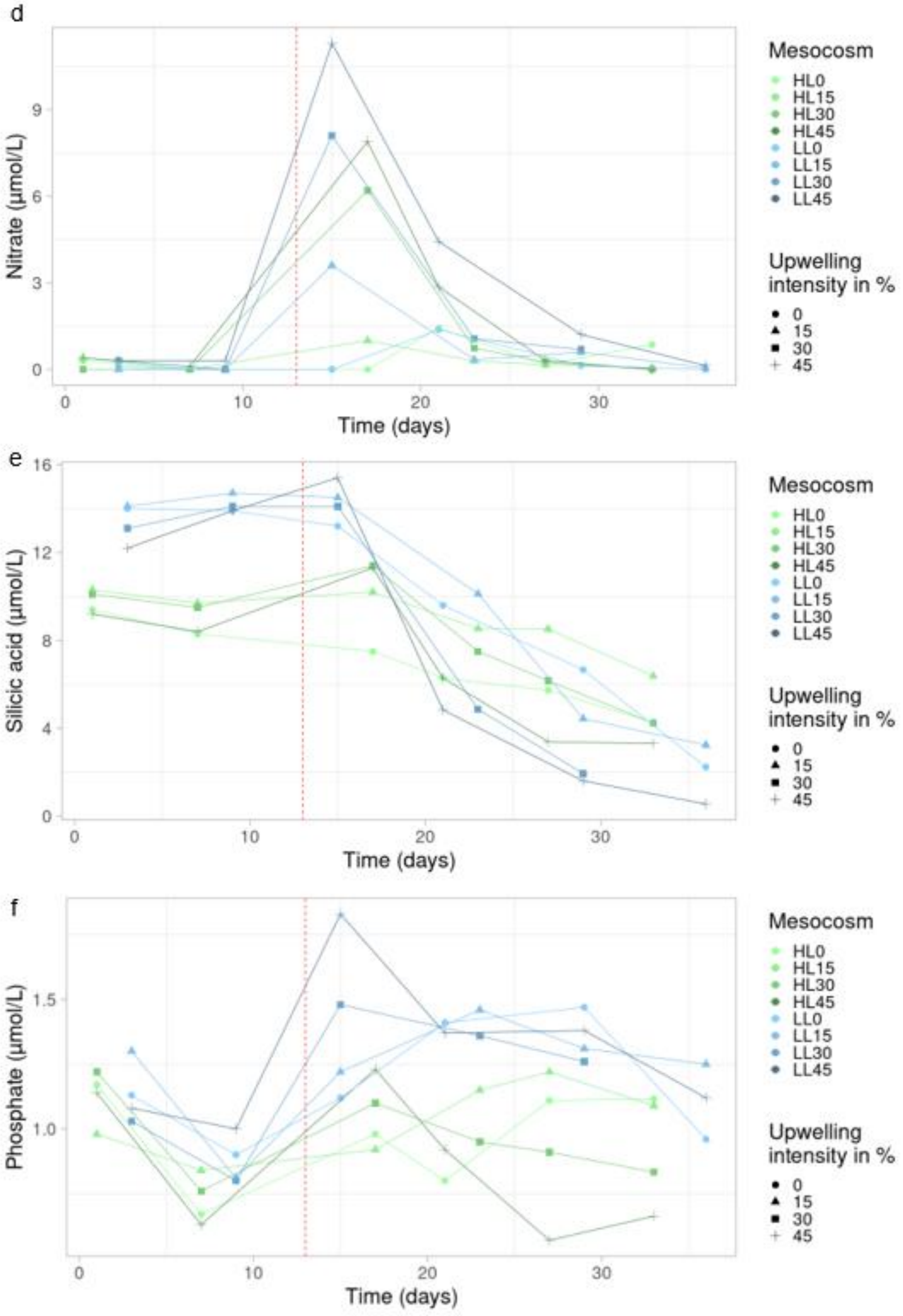

Supplementary file 4. ASV-to-sample matrix with assigned taxonomy for the samples of the eight mesocosms. Can be accessed through the following link: https://seafile.rlp.net/d/fc24d0c264e447eda5ad/.

Supplementary file 5. Sequence data overview from raw sequence files to high-quality sequences used in downstream analyses for all samples of the eight mesocosms under study. Supplied as pdf.

Supplementary file 6. Rarefaction profiles for all samples of the eight mesocosms under study for 0%, 15%, 30% and 45% upwelling intensity. Profiles show (near-)sample saturation for all samples. Supplied as pdf file.

Supplementary file 7. ASV richness, evenness, Shannon Index and Simpson Index for each sample. Supplied as excel file.

Supplementary file 8. Shapiro Wilk and Levene´s test results for each alpha diversity measurement. Verification of the normal distribution and variance homogeneity for ASV richness, evenness, Shannon and Simpson Index.

**Model**	**Test**	**p-value**
ASV richness	Shapiro Wilk	0.730
	Levene	0.513
Evenness	Shapiro Wilk	0.351
	Levene	0.606
Shannon Index H´	Shapiro Wilk	0.398
	Levene	0.617
Simpson Index D	Shapiro Wilk	0.101
	Levene	0.827

Supplementary file 9. PERMANOVA analysis on Bray-Curtis distances. PERMANOVA was performed to test significant effects for mesocosm manipulations. DF: degree of freedom; SS: sum of squares; F: F-statistic.

**PERMANOVA**	**DF**	**SS**	**F**	**R^2**	**p-value**

Light treatment	1	0.92	3.95	0.07	>0.001
Upwelling intensity	3	1.05	4.55	0.08	>0.001

Supplementary file 10. Correlation of the environmental parameters to the NMDS ordination. *Envfit* results of the physicochemical parameters. R^2: coefficient of determination; PAR: photosynthetically active radiation.

**Parameter**	**NMDS1**	**NMDS2**	**R^2**	**p-value**
Temperature	-0.49	-0.13	0.26	0.001
Salinity	0.62	0.18	0.41	0.0001
Density	0.63	0.17	0.42	0.0001
pH	-0.17	0.62	0.42	0.0001
Dissolved oxygen	-0.45	0.44	0.40	0.0001
Chlorophyll a	-0.77	0.18	0.62	0.0001
PAR	-0.03	0.22	0.05	0.3265
Nitrate	0.07	-0.09	0.01	0.7704
Nitrite	0.02	-0.27	0.07	0.1862
Phosphate	0.22	-0.70	0.54	0.0001
Orthosilicic acid	-0.64	-0.33	0.52	0.0001
Extern	-0.68	-0.13	0.48	0.0001
Intern	-0.83	0.15	0.72	0.0001
No Ingestion	0.23	-0.15	0.07	0.1726
Osmotrophic	0.33	-0.70	0.60	0.0001
Saprotrophic	0.32	0.23	0.16	0.0227
Unknown Ingestion	0.09	0.64	0.42	0.0001
Commensalist	0.44	-0.06	0.20	0.0055
Mutualist Photosynthetic	-0.12	0.49	0.25	0.0013
No Symbiosis	-0.50	0.08	0.25	0.0015
Parasite	0.09	-0.38	0.15	0.0252
Unknown Symbiosis	0.46	-0.44	0.41	0.0001

Supplementary file 11. Changes in relative read abundance of the dominant divisions as line plot for the top 5 divisions across key treatments to illustrate temporal trajectories.

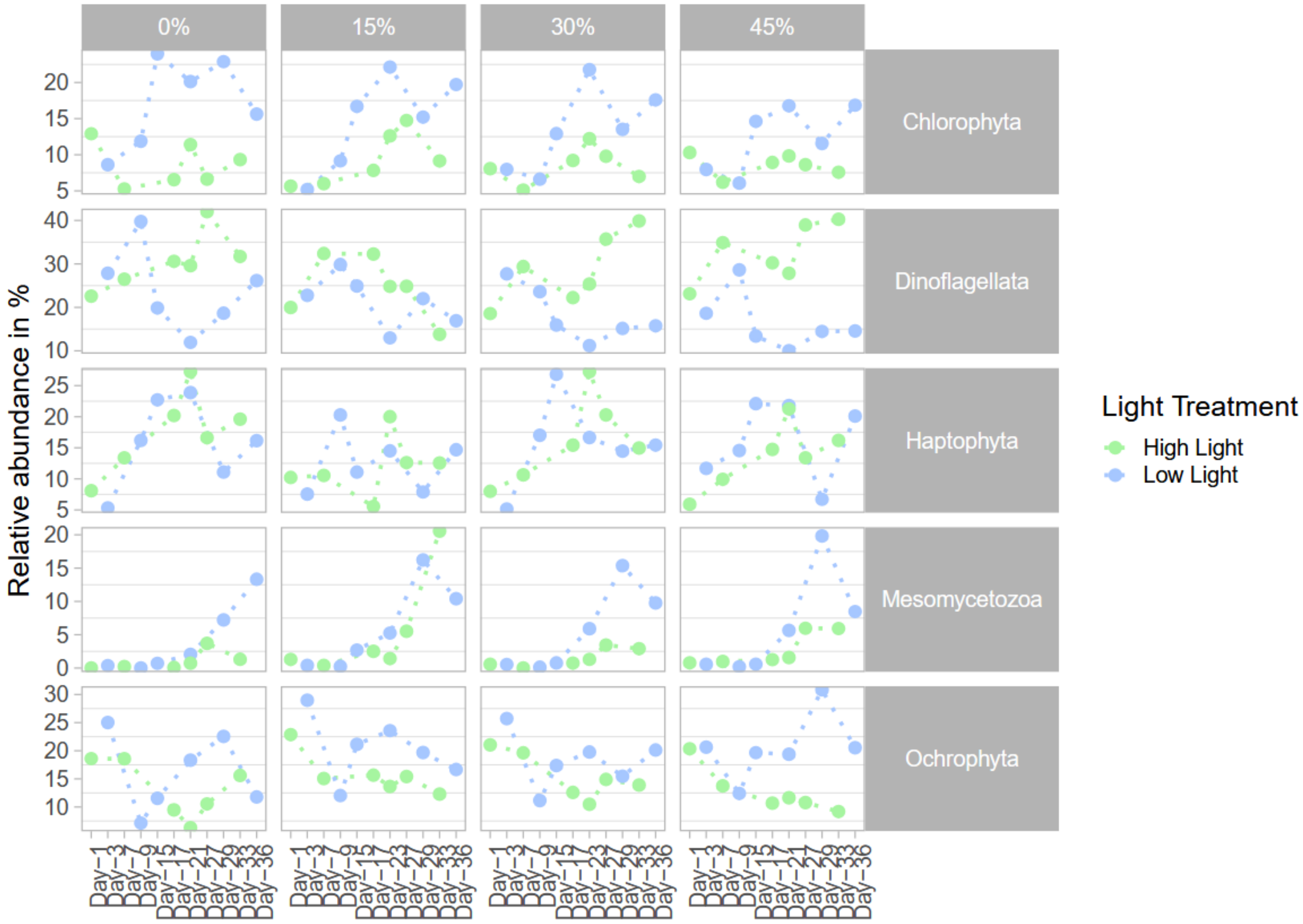

Supplement: Supplementary file 1 — File S1: ece372827‐sup‐0001‐FileS1.pdf. [file ECE3-16-e72827-s004.pdf]
