# Supplementary material for: Protistan Plankton Responses to Variable Light and Upwelling in the Peruvian Humboldt Current System: Insights Into Community Dynamics Under Environmental Change
Source: Ecol Evol. 2026 Jan 12;16(1):e72827. doi: 10.1002/ece3.72827 (PMC12796512; doi:10.1002/ece3.72827)
Supplement: Supplementary file 2 — File S2: ece372827‐sup‐0002‐FileS2.pdf. [file ECE3-16-e72827-s005.pdf]

sample,Mesokosmos,light,upwelling intensity in %,days,time,Temperature in °C,Salinity in PSU,Densi
High_Light_0._Deep_Water_Day_1,1,high,0,1,02.28.2020,18.03,35.01,25.29,8.08,6.46,84.92,24.37,5
High_Light_15._Deep_Water_Day_1,7,high,15,1,02.28.2020,18.26,35.00,25.23,8.14,6.82,90.02,20.4
High_Light_30._Deep_Water_Day_1,3,high,30,1,02.28.2020,18.08,35.01,25.28,8.09,6.36,83.81,24.0
High_Light_45._Deep_Water_Day_1,5,high,45,1,02.28.2020,18.20,35.01,25.25,8.10,6.52,86.01,23.0
Low_Light_0._Deep_Water_Day_2,8,low,0,3,03.01.2020,18.24,35.00,25.23,8.04,6.25,82.28,21.06,57
Low_Light_15._Deep_Water_Day_2,6,low,15,3,03.01.2020,18.29,35.00,25.22,8.06,6.20,81.68,19.32
Low_Light_30._Deep_Water_Day_2,4,low,30,3,03.01.2020,18.26,35.00,25.23,8.03,6.36,83.74,20.90
Low_Light_45._Deep_Water_Day_2,2,low,45,3,03.01.2020,18.38,35.00,25.20,8.05,6.22,82.08,22.01
High_Light_0._Deep_Water_Day_3,1,high,0,7,03.05.2020,18.47,35.13,25.28,8.34,7.07,93.35,16.76,9
High_Light_15._Deep_Water_Day_3,7,high,15,7,03.05.2020,18.35,35.14,25.31,8.31,6.99,92.23,12.3
High_Light_30._Deep_Water_Day_3,3,high,30,7,03.05.2020,18.46,35.14,25.29,8.34,7.22,95.27,16.2
High_Light_45._Deep_Water_Day_3,5,high,45,7,03.05.2020,18.49,35.14,25.28,8.33,7.53,99.44,19.9
Low_Light_0._Deep_Water_Day_4,8,low,0,9,03.07.2020,17.81,35.18,25.48,8.11,5.09,66.61,4.40,305
Low_Light_15._Deep_Water_Day_4,6,low,15,9,03.07.2020,17.83,35.18,25.47,8.14,5.20,68.02,4.60,9
Low_Light_30._Deep_Water_Day_4,4,low,30,9,03.07.2020,17.76,35.19,25.50,8.13,5.30,69.18,4.84,6
Low_Light_45._Deep_Water_Day_4,2,low,45,9,03.07.2020,17.87,35.16,25.45,8.06,4.59,60.25,4.19,5
Low_Light_0._Deep_Water_Day_5,8,low,0,15,03.13.2020,17.98,35.20,25.45,8.04,4.87,63.93,9.91,17
Low_Light_15._Deep_Water_Day_5,6,low,15,15,03.13.2020,18.09,35.18,25.41,8.00,5.27,69.39,11.4
Low_Light_30._Deep_Water_Day_5,4,low,30,15,03.13.2020,17.99,35.18,25.43,7.93,5.16,67.69,8.08
Low_Light_45._Deep_Water_Day_5,2,low,45,15,03.13.2020,18.01,35.13,25.39,7.84,5.48,71.79,9.03
High_Light_0._Deep_Water_Day_6,1,high,0,17,03.15.2020,17.40,35.18,25.58,8.24,6.34,82.03,9.62,7
High_Light_15._Deep_Water_Day_6,7,high,15,17,03.15.2020,17.47,35.17,25.56,8.17,6.45,83.72,10.
High_Light_30._Deep_Water_Day_6,3,high,30,17,03.15.2020,17.44,35.15,25.55,8.10,6.70,86.86,11.
High_Light_45._Deep_Water_Day_6,5,high,45,17,03.15.2020,17.48,35.14,25.53,8.04,6.64,86.12,12.
High_Light_0._Deep_Water_Day_7,1,high,0,21,03.19.2020,17.98,35.17,25.43,8.19,6.01,78.78,6.19,9
High_Light_45._Deep_Water_Day_7,5,high,45,21,03.19.2020,18.09,35.13,25.37,8.12,6.46,84.77,15.
Low_Light_0._Deep_Water_Day_7,8,low,0,21,03.19.2020,18.07,35.19,25.43,8.00,5.35,70.27,4.94,16
Low_Light_45._Deep_Water_Day_7,2,low,45,21,03.19.2020,18.02,35.11,25.38,7.95,5.87,76.86,7.51
High_Light_15._Deep_Water_Day_8,7,high,15,23,03.21.2020,17.35,35.16,25.58,8.12,5.83,75.45,3.8
High_Light_30._Deep_Water_Day_8,3,high,30,23,03.21.2020,17.34,35.14,25.56,8.14,6.08,78.64,8.6
Low_Light_15._Deep_Water_Day_8,6,low,15,23,03.21.2020,17.38,35.17,25.57,8.00,5.72,74.06,4.42
Low_Light_30._Deep_Water_Day_8,4,low,30,23,03.21.2020,17.31,35.16,25.59,8.00,5.70,73.68,7.52
High_Light_0._Deep_Water_Day_9,1,high,0,27,03.25.2020,17.84,35.19,25.48,8.13,5.68,74.31,5.02,5
High_Light_15._Deep_Water_Day_9,7,high,15,27,03.25.2020,17.91,35.16,25.44,8.10,5.85,76.64,3.1
High_Light_30._Deep_Water_Day_9,3,high,30,27,03.25.2020,17.85,35.14,25.44,8.11,5.83,76.34,8.3
High_Light_45._Deep_Water_Day_9,5,high,45,27,03.25.2020,17.86,35.12,25.42,8.16,6.16,80.61,18.
Low_Light_0._Deep_Water_Day_10,8,low,0,29,03.27.2020,17.89,35.19,25.47,8.02,5.49,71.85,3.14,
Low_Light_15._Deep_Water_Day_10,6,low,15,29,03.27.2020,17.91,35.16,25.44,8.05,5.72,74.86,3.5
Low_Light_30._Deep_Water_Day_10,4,low,30,29,03.27.2020,17.93,35.17,25.44,8.04,5.55,72.63,3.6
Low_Light_45._Deep_Water_Day_10,2,low,45,29,03.27.2020,17.92,35.12,25.41,8.01,5.49,71.81,3.8
High_Light_0._Deep_Water_Day_11,1,high,0,33,03.31.2020,17.86,35.20,25.48,8.19,5.46,71.53,5.19,
High_Light_15._Deep_Water_Day_11,7,high,15,33,03.31.2020,17.81,35.17,25.47,8.21,5.99,78.25,5.
High_Light_30._Deep_Water_Day_11,3,high,30,33,03.31.2020,17.86,35.14,25.44,8.24,6.05,79.29,7.
High_Light_45._Deep_Water_Day_11,5,high,45,33,03.31.2020,17.85,35.13,25.43,8.22,5.23,68.42,6.
Low_Light_0._Deep_Water_Day_12,8,low,0,36,04.02.2020,17.71,35.20,25.52,8.07,5.05,65.94,1.91,
Low_Light_15._Deep_Water_Day_12,6,low,15,36,04.02.2020,17.82,35.18,25.48,8.11,5.50,71.90,2.6
Low_Light_30._Deep_Water_Day_12,4,low,30,36,04.02.2020,17.70,35.18,25.51,8.08,5.14,66.99,2.9
Low_Light_45._Deep_Water_Day_12,2,low,45,36,04.02.2020,17.82,35.14,25.45,8.08,5.50,71.90,3.9

ity in kg m3,pH,Dissolved oxygen in mg L-1,Oxygen saturation in %,Chlorophyll-a in Âµg L-1,PAR in Âµ

.mol photons m2 s-1,Nitrate Âµmol L-1,Nitrite Âµmol L-1,TotalN Âµmol L-1,Phosphate Âµmol L-1,Silic

:cate Âµmol L-1
