# Supplementary material for: Protistan Plankton Responses to Variable Light and Upwelling in the Peruvian Humboldt Current System: Insights Into Community Dynamics Under Environmental Change
Source: Ecol Evol. 2026 Jan 12;16(1):e72827. doi: 10.1002/ece3.72827 (PMC12796512; doi:10.1002/ece3.72827)
Supplement: Supplementary file 3 — File S3: ece372827‐sup‐0003‐FileS3.pdf. [file ECE3-16-e72827-s003.pdf]

sample_name	Replicate	input	filtered	Denoised & dereplicatedF
High_Light_0%_Deep_Water_Order_1	1	444693	425002	249328
High_Light_0%_Deep_Water_Order_1	2	176277	169161	104123
High_Light_0%_Deep_Water_Order_1	3	131846	127003	78768
High_Light_0%_Deep_Water_Order_11	1	142456	135916	89634
High_Light_0%_Deep_Water_Order_11	2	162757	155602	113159
High_Light_0%_Deep_Water_Order_11	3	578816	548236	373965
High_Light_0%_Deep_Water_Order_3	1	164481	157623	104805
High_Light_0%_Deep_Water_Order_3	2	111528	106871	65397
High_Light_0%_Deep_Water_Order_3	3	541542	514359	318814
High_Light_0%_Deep_Water_Order_6	1	571227	542682	366082
High_Light_0%_Deep_Water_Order_6	2	521238	495116	338582
High_Light_0%_Deep_Water_Order_6	3	386086	367879	254505
High_Light_0%_Deep_Water_Order_7	1	559852	526369	390168
High_Light_0%_Deep_Water_Order_7	2	510587	482157	390524
High_Light_0%_Deep_Water_Order_7	3	565463	532203	414485
High_Light_0%_Deep_Water_Order_9	1	331719	315104	228760
High_Light_0%_Deep_Water_Order_9	2	370266	354982	237551
High_Light_0%_Deep_Water_Order_9	3	111928	107053	77056
Low_Light_45%_Deep_Water_Order_10	1	317531	297652	187084
Low_Light_45%_Deep_Water_Order_10	2	433554	408254	266182
Low_Light_45%_Deep_Water_Order_10	3	500955	472565	319111
Low_Light_45%_Deep_Water_Order_12	1	494663	464826	335554
Low_Light_45%_Deep_Water_Order_12	2	429712	404464	283094
Low_Light_45%_Deep_Water_Order_12	3	158914	151012	122974
Low_Light_45%_Deep_Water_Order_2	1	150361	143300	87544
Low_Light_45%_Deep_Water_Order_2	2	265174	253458	162583
Low_Light_45%_Deep_Water_Order_2	3	53941	51755	32696
Low_Light_45%_Deep_Water_Order_4	1	126580	121192	85341
Low_Light_45%_Deep_Water_Order_4	2	177651	170308	109040
Low_Light_45%_Deep_Water_Order_4	3	371468	353303	227580
Low_Light_45%_Deep_Water_Order_5	1	590494	555806	474478
Low_Light_45%_Deep_Water_Order_5	2	586538	553946	399382
Low_Light_45%_Deep_Water_Order_5	3	444605	421024	316368
Low_Light_45%_Deep_Water_Order_7	1	513826	483354	324151
Low_Light_45%_Deep_Water_Order_7	2	1014554	953361	695876
Low_Light_45%_Deep_Water_Order_7	3	241830	230257	153805
High_Light_30%_Deep_Water_Order_1	1	217782	208977	128845
High_Light_30%_Deep_Water_Order_1	2	280676	267736	158149
High_Light_30%_Deep_Water_Order_1	3	594828	568162	356682
High_Light_30%_Deep_Water_Order_11	1	597038	571757	357026
High_Light_30%_Deep_Water_Order_11	2	417033	401255	251111
High_Light_30%_Deep_Water_Order_11	3	343636	327929	230689
High_Light_30%_Deep_Water_Order_3	1	359256	341589	215776
High_Light_30%_Deep_Water_Order_3	2	380335	363751	236404
High_Light_30%_Deep_Water_Order_3	3	315256	303374	181825
High_Light_30%_Deep_Water_Order_6	1	491951	471878	334439
High_Light_30%_Deep_Water_Order_6	2	1023081	976006	651790
High_Light_30%_Deep_Water_Order_6	3	484830	465527	306617
High_Light_30%_Deep_Water_Order_8	1	150323	143140	104001
High_Light_30%_Deep_Water_Order_8	2	222796	212946	160315
High_Light_30%_Deep_Water_Order_8	3	378809	358641	271726
High_Light_30%_Deep_Water_Order_9	1	181966	174950	126862

High_Light_30%_Deep_Water_Order_9	2	557117	530864	357455
High_Light_30%_Deep_Water_Order_9	3	480069	458471	293147
Low_Light_30%_Deep_Water_Order_10	1	364868	345987	243085
Low_Light_30%_Deep_Water_Order_10	2	491322	468253	328523
Low_Light_30%_Deep_Water_Order_10	3	600019	570305	411104
Low_Light_30%_Deep_Water_Order_12	1	539096	509301	367852
Low_Light_30%_Deep_Water_Order_12	2	175521	164684	111551
Low_Light_30%_Deep_Water_Order_12	3	240156	227426	172873
Low_Light_30%_Deep_Water_Order_2	1	772225	738833	421026
Low_Light_30%_Deep_Water_Order_2	2	521917	502094	299715
Low_Light_30%_Deep_Water_Order_2	3	344783	330425	199476
Low_Light_30%_Deep_Water_Order_4	1	796334	761623	518106
Low_Light_30%_Deep_Water_Order_4	2	467912	449911	298096
Low_Light_30%_Deep_Water_Order_4	3	337153	320097	224079
Low_Light_30%_Deep_Water_Order_5	1	208332	195590	155161
Low_Light_30%_Deep_Water_Order_5	2	641848	603417	468394
Low_Light_30%_Deep_Water_Order_5	3	286410	268144	231980
Low_Light_30%_Deep_Water_Order_8	1	411367	388103	264778
Low_Light_30%_Deep_Water_Order_8	2	379281	357849	230692
Low_Light_30%_Deep_Water_Order_8	3	461628	438707	298478
High_Light_45%_Deep_Water_Order_1	1	80937	77548	46578
High_Light_45%_Deep_Water_Order_1	2	346103	329998	193932
High_Light_45%_Deep_Water_Order_1	3	57953	55200	33846
High_Light_45%_Deep_Water_Order_11	1	70622	67007	48774
High_Light_45%_Deep_Water_Order_11	2	637912	608862	390053
High_Light_45%_Deep_Water_Order_11	3	329110	315028	208171
High_Light_45%_Deep_Water_Order_3	1	436096	416413	246245
High_Light_45%_Deep_Water_Order_3	2	379389	363072	205577
High_Light_45%_Deep_Water_Order_3	3	870228	836535	486696
High_Light_45%_Deep_Water_Order_6	1	412597	396695	256120
High_Light_45%_Deep_Water_Order_6	2	575443	550345	392200
High_Light_45%_Deep_Water_Order_6	3	204548	196982	125636
High_Light_45%_Deep_Water_Order_7	1	476872	456332	316089
High_Light_45%_Deep_Water_Order_7	2	500260	479934	369100
High_Light_45%_Deep_Water_Order_7	3	210737	200379	151750
High_Light_45%_Deep_Water_Order_9	1	404663	392356	235162
High_Light_45%_Deep_Water_Order_9	2	637407	608486	371731
High_Light_45%_Deep_Water_Order_9	3	600769	575360	388930
Low_Light_15%_Deep_Water_Order_10	1	138675	130389	89736
Low_Light_15%_Deep_Water_Order_10	2	211453	197561	137649
Low_Light_15%_Deep_Water_Order_10	3	495600	464686	294509
Low_Light_15%_Deep_Water_Order_12	1	299796	287015	205621
Low_Light_15%_Deep_Water_Order_12	2	650269	617278	415982
Low_Light_15%_Deep_Water_Order_12	3	479674	456683	296530
Low_Light_15%_Deep_Water_Order_2	1	590273	561655	325675
Low_Light_15%_Deep_Water_Order_2	2	619572	594848	336834
Low_Light_15%_Deep_Water_Order_2	3	1066868	1E+06	608413
Low_Light_15%_Deep_Water_Order_4	1	289812	277023	189628
Low_Light_15%_Deep_Water_Order_4	2	411738	395586	259550
Low_Light_15%_Deep_Water_Order_4	3	149882	142343	100535
Low_Light_15%_Deep_Water_Order_5	1	839145	799893	508263
Low_Light_15%_Deep_Water_Order_5	2	369159	352827	215524
Low_Light_15%_Deep_Water_Order_5	3	154598	147543	92555

Low_Light_15%_Deep_Water_Order_8	1	329971	312156	222473
Low_Light_15%_Deep_Water_Order_8	2	107447	101395	70580
Low_Light_15%_Deep_Water_Order_8	3	124599	117421	78566
High_Light_15%_Deep_Water_Order_1	1	132036	127227	77755
High_Light_15%_Deep_Water_Order_1	2	247477	235076	144833
High_Light_15%_Deep_Water_Order_1	3	172058	164035	111072
High_Light_15%_Deep_Water_Order_11	1	612868	582708	466854
High_Light_15%_Deep_Water_Order_11	2	566882	538988	376812
High_Light_15%_Deep_Water_Order_11	3	228953	218424	152366
High_Light_15%_Deep_Water_Order_3	1	209745	200881	134625
High_Light_15%_Deep_Water_Order_3	2	105541	101219	63390
High_Light_15%_Deep_Water_Order_3	3	322029	308536	181018
High_Light_15%_Deep_Water_Order_6	1	165689	158862	95617
High_Light_15%_Deep_Water_Order_6	2	235424	225033	133862
High_Light_15%_Deep_Water_Order_6	3	275400	261747	179572
High_Light_15%_Deep_Water_Order_8	1	421629	398354	278114
High_Light_15%_Deep_Water_Order_8	2	594535	559644	458062
High_Light_15%_Deep_Water_Order_8	3	284928	270439	200235
High_Light_15%_Deep_Water_Order_9	1	310414	291966	205009
High_Light_15%_Deep_Water_Order_9	2	240370	227693	150897
High_Light_15%_Deep_Water_Order_9	3	363585	346685	221422
Low_Light_0%_Deep_Water_Order_10	1	181986	171279	110198
Low_Light_0%_Deep_Water_Order_10	2	138372	129984	80899
Low_Light_0%_Deep_Water_Order_10	3	367247	338203	225057
Low_Light_0%_Deep_Water_Order_12	1	184269	175368	117220
Low_Light_0%_Deep_Water_Order_12	2	961501	911222	602995
Low_Light_0%_Deep_Water_Order_12	3	639389	605844	388358
Low_Light_0%_Deep_Water_Order_2	1	674975	651307	373456
Low_Light_0%_Deep_Water_Order_2	2	385984	369121	216385
Low_Light_0%_Deep_Water_Order_2	3	292364	279769	172335
Low_Light_0%_Deep_Water_Order_4	1	177924	170312	111622
Low_Light_0%_Deep_Water_Order_4	2	56618	54183	36283
Low_Light_0%_Deep_Water_Order_4	3	32973	31483	20268
Low_Light_0%_Deep_Water_Order_5	1	142042	133850	99948
Low_Light_0%_Deep_Water_Order_5	2	382847	358752	276559
Low_Light_0%_Deep_Water_Order_5	3	453450	425942	332029
Low_Light_0%_Deep_Water_Order_7	1	1000598	950152	804192
Low_Light_0%_Deep_Water_Order_7	2	810678	766483	598567
Low_Light_0%_Deep_Water_Order_7	3	367690	349960	265087

Denoised and dereplicatedR	merged	ASVs_postCC	reads_postCC	target ASVs	target reads
274931	230681	1244	230418	1222	229813
118199	99415	572	99323	566	99253
88640	74624	542	74518	536	74423
116580	86956	584	86930	549	84116
139631	110019	622	109977	582	105378
470661	363685	1844	363536	1770	351388
112903	96750	906	96438	889	96013
72447	56636	525	56558	515	56420
362467	287495	2048	286687	2019	284955
406315	346131	1693	346013	1578	316145
373599	316619	1554	316573	1451	286073
275615	240508	1077	240488	1007	219947
437511	377715	1597	377686	1397	293646
418086	378249	1398	378218	1160	273406
456909	400822	1676	400801	1449	300902
295732	223631	967	223475	893	176144
316321	230626	1360	230062	1327	218435
95321	73864	366	73836	336	61234
285238	181335	1000	181078	974	180117
365071	231395	1190	230826	1147	229135
433918	310994	1579	310267	1528	306618
389669	324686	1387	324614	1309	311618
324299	273962	1136	273911	1066	260074
132839	120237	509	120231	448	111965
107611	74989	750	74934	707	74093
184811	152929	1072	152718	1047	151909
35473	30510	331	30500	319	30330
90611	80136	578	80081	542	73157
116318	99662	710	99647	680	96782
256206	196063	1277	195794	1219	179646
506677	465969	1542	465959	1373	392383
483062	380777	1648	380742	1503	347291
371586	292870	1387	292851	1282	262266
389258	302456	1505	302401	1394	276850
784259	598028	2988	597701	2801	531893
167580	148487	753	147995	709	143434
139253	118542	784	118347	770	118141
182839	146545	927	146333	912	146051
425370	338606	1913	338097	1896	337895
380253	337396	2027	337262	1972	324959
283245	239264	1362	239151	1307	229137
243279	220239	1204	220183	1150	209692
234226	197694	1558	197059	1532	195574
250344	213612	1562	213084	1541	212133
202969	163837	1392	163240	1373	162649
365226	308884	1392	308837	1212	229281
761097	600276	2527	600231	2268	492030
349887	273450	1345	273444	1222	227397
112181	99495	607	99486	560	88997
170597	154125	892	154040	834	140372
291233	261617	1199	261567	1110	231655
133919	122289	761	122171	726	114239

381570	342617	1946	342389	1864	308476
305495	275916	1694	275732	1626	257492
292106	234712	979	234652	905	220809
414267	322240	1124	322146	1048	309449
496535	402465	1462	402357	1369	378877
448991	359827	1356	359628	1290	346180
142118	105893	674	105878	632	101111
199258	168297	675	168188	627	161292
463338	355694	2298	355422	2292	355189
323821	275578	1323	275533	1320	275429
215018	188308	1220	187923	1200	187408
556350	482266	2598	481778	2501	452315
327522	263958	1467	263858	1407	251124
231782	209376	1002	209357	943	191310
162580	149528	696	149371	592	111632
524736	451281	2158	449901	1835	342347
242915	224549	1154	223956	928	154061
303857	255478	1108	255322	1028	239821
307468	222619	1118	222575	1036	209716
344923	291175	1133	291123	1043	268571
51643	41425	394	41362	384	41246
211491	183527	1156	183097	1127	182387
38151	31404	322	31404	308	31180
51288	46953	408	46940	377	42370
411007	367286	2302	367099	2210	333723
217816	197390	1336	197321	1292	189404
254588	228660	827	228655	810	226323
218364	152500	861	152409	839	151515
535028	441997	1651	441659	1622	439141
305456	245150	1150	245126	1054	210451
436012	373624	1520	373565	1366	297053
146625	114850	1063	114607	1001	107952
355295	298862	1578	298818	1468	225966
395718	357670	1382	357648	1218	234573
169168	147150	703	147150	598	81687
239046	213399	1823	213125	1791	211742
384839	347131	2982	346939	2941	340283
400740	368536	3012	367213	2943	352240
117899	86779	466	86731	451	86265
180639	134568	706	134444	676	133134
409133	285930	1380	285748	1347	282345
231478	199268	713	199060	653	186087
497023	402542	1498	402246	1416	375975
360867	287359	1040	287227	970	273678
355884	295118	1628	294969	1591	293411
369373	298879	1611	298600	1570	297041
664458	573149	2605	572680	2552	568347
198431	178742	824	178696	763	164900
275252	243457	1247	243155	1176	226968
103305	93747	468	93719	435	82537
557057	468675	1717	468500	1616	424250
250283	197781	983	197460	951	194614
116221	85879	518	85824	494	83748

264802	216532	1025	216003	951	204968
80691	67916	448	67907	405	63795
95088	75485	484	75412	438	71858
84780	72700	662	72696	622	71259
167744	138034	899	137948	871	137352
118817	105309	782	105307	736	103420
513574	452080	1529	452079	1425	413795
453356	360082	1570	359492	1525	353581
184004	142506	853	142499	807	136734
144923	127928	746	127894	703	125992
67441	59289	450	59288	420	58578
196913	148396	900	148358	864	146806
120768	88024	672	87916	648	86950
175366	126036	818	125784	785	123296
213357	171319	1144	171159	944	106892
344838	250501	1145	250440	1030	179906
514393	438493	1361	438431	1033	244564
240183	192271	860	192259	764	124452
249525	198172	1101	198044	1048	189739
191078	141488	863	141411	819	135521
296368	212991	1187	212835	1135	204751
137282	107013	458	106791	435	104577
101326	76675	506	76653	468	73417
289592	218663	1059	218415	1013	214110
126201	111515	632	111498	594	107543
648740	581679	2296	581380	2174	551788
415270	371563	1564	371387	1487	359743
404192	331601	2185	330305	2177	330235
228392	200202	1240	199926	1220	199470
184338	158616	1181	158290	1166	157918
114166	104061	487	103953	457	97455
37068	33545	273	33514	256	31146
20288	17685	167	17685	159	16881
103362	93509	498	93303	413	68882
283162	265312	1141	264759	912	179329
344098	316212	1083	316165	877	216885
854915	782589	2243	782293	1961	640488
664051	580808	2187	580514	1968	479503
285291	254742	944	254636	835	211942

sample_name	Time in days	date
High_Light_0._Deep_Water_Order_1	1	02.28.2020
High_Light_0._Deep_Water_Order_3	7	03.05.2020
High_Light_0._Deep_Water_Order_6	17	03.15.2020
High_Light_0._Deep_Water_Order_7	21	03.19.2020
High_Light_0._Deep_Water_Order_9	27	03.25.2020
High_Light_0._Deep_Water_Order_11	33	03.31.2020
High_Light_15._Deep_Water_Order_1	1	02.28.2020
High_Light_15._Deep_Water_Order_3	7	03.05.2020
High_Light_15._Deep_Water_Order_6	17	03.15.2020
High_Light_15._Deep_Water_Order_8	23	03.21.2020
High_Light_15._Deep_Water_Order_9	27	03.25.2020
High_Light_15._Deep_Water_Order_11	33	03.31.2020
High_Light_30._Deep_Water_Order_1	1	02.28.2020
High_Light_30._Deep_Water_Order_3	7	03.05.2020
High_Light_30._Deep_Water_Order_6	17	03.15.2020
High_Light_30._Deep_Water_Order_8	23	03.21.2020
High_Light_30._Deep_Water_Order_9	27	03.25.2020
High_Light_30._Deep_Water_Order_11	33	03.31.2020
High_Light_45._Deep_Water_Order_1	1	02.28.2020
High_Light_45._Deep_Water_Order_3	7	03.05.2020
High_Light_45._Deep_Water_Order_6	17	03.15.2020
High_Light_45._Deep_Water_Order_7	21	03.19.2020
High_Light_45._Deep_Water_Order_9	27	03.25.2020
High_Light_45._Deep_Water_Order_11	33	03.31.2020
Low_Light_0._Deep_Water_Order_10	29	03.27.2020
Low_Light_0._Deep_Water_Order_12	36	04.02.2020
Low_Light_0._Deep_Water_Order_2	3	03.01.2020
Low_Light_0._Deep_Water_Order_4	9	03.07.2020
Low_Light_0._Deep_Water_Order_5	15	03.13.2020
Low_Light_0._Deep_Water_Order_7	21	03.19.2020
Low_Light_15._Deep_Water_Order_10	29	03.27.2020
Low_Light_15._Deep_Water_Order_12	36	04.02.2020
Low_Light_15._Deep_Water_Order_2	3	03.01.2020
Low_Light_15._Deep_Water_Order_4	9	03.07.2020
Low_Light_15._Deep_Water_Order_5	15	03.13.2020
Low_Light_15._Deep_Water_Order_8	23	03.21.2020
Low_Light_30._Deep_Water_Order_10	29	03.27.2020
Low_Light_30._Deep_Water_Order_12	36	04.02.2020
Low_Light_30._Deep_Water_Order_2	3	03.01.2020
Low_Light_30._Deep_Water_Order_4	9	03.07.2020
Low_Light_30._Deep_Water_Order_5	15	03.13.2020
Low_Light_30._Deep_Water_Order_8	23	03.21.2020
Low_Light_45._Deep_Water_Order_10	29	03.27.2020
Low_Light_45._Deep_Water_Order_12	36	04.02.2020
Low_Light_45._Deep_Water_Order_2	3	03.01.2020
Low_Light_45._Deep_Water_Order_4	9	03.07.2020
Low_Light_45._Deep_Water_Order_5	15	03.13.2020
Low_Light_45._Deep_Water_Order_7	21	03.19.2020
