# Supplementary material for: Protistan Plankton Responses to Variable Light and Upwelling in the Peruvian Humboldt Current System: Insights Into Community Dynamics Under Environmental Change
Source: Ecol Evol. 2026 Jan 12;16(1):e72827. doi: 10.1002/ece3.72827 (PMC12796512; doi:10.1002/ece3.72827)
Supplement: Supplementary file 4 — File S4: ece372827‐sup‐0004‐FileS4.pdf. [file ECE3-16-e72827-s002.pdf]

Supplementary file 6. Rarefaction profiles for all samples of the eight mesocosms under study for 0%, 15%, 30% and 45% upwelling intensity. Profiles show (near-)sample saturation for all samples.

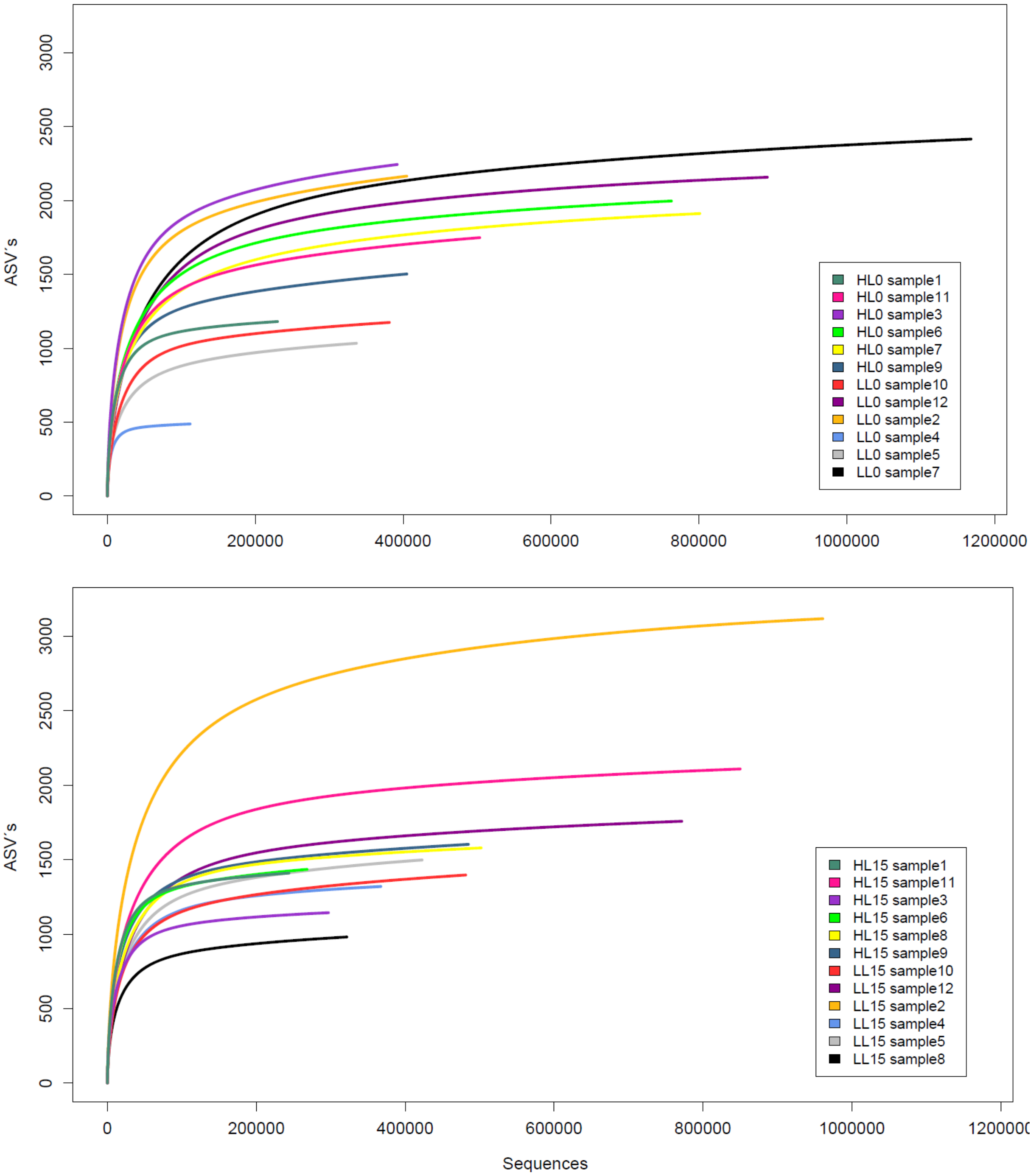

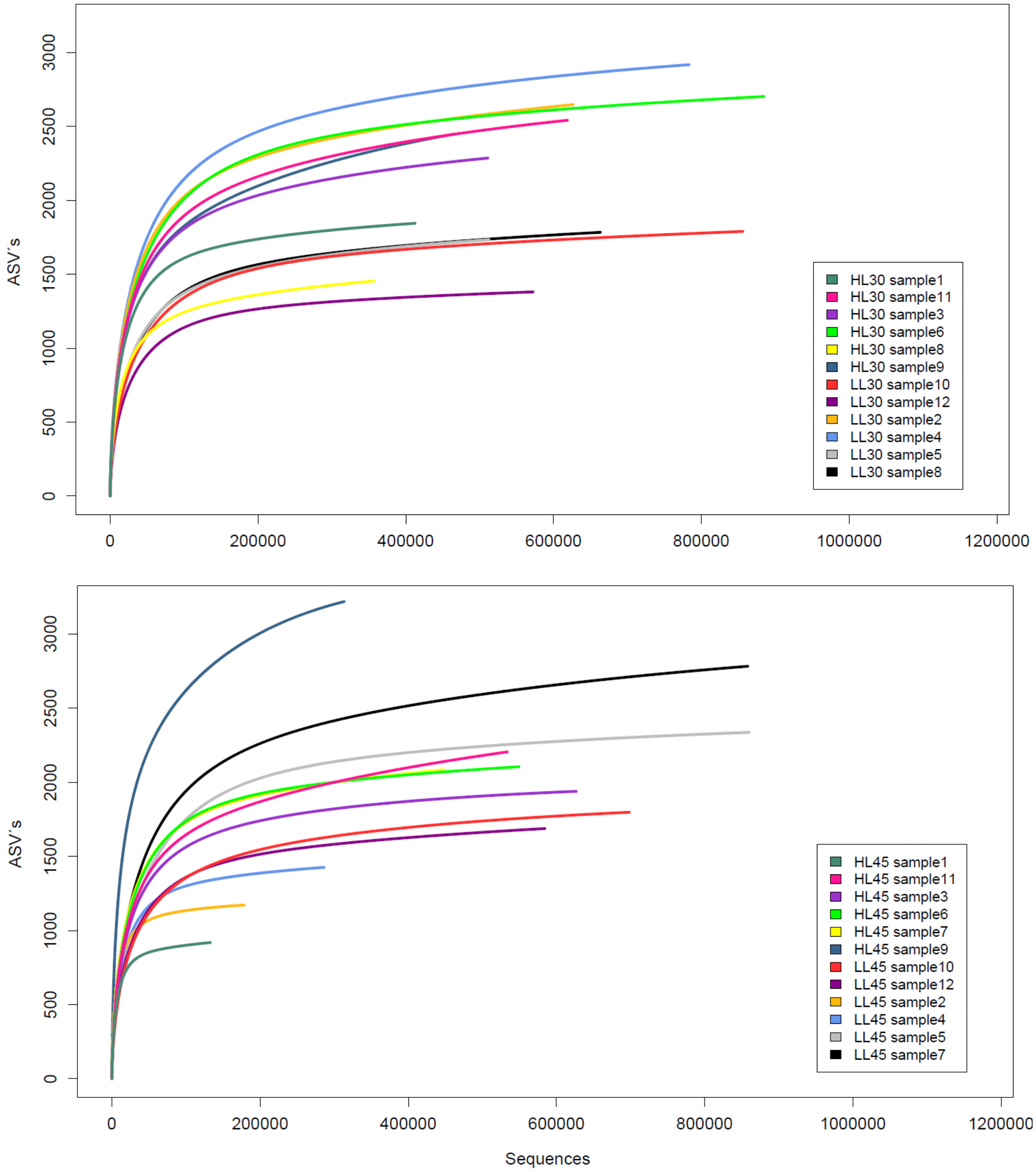
ASV´s
0
500
1000
1500
2000
2500
3000
0
200000
400000
600000
800000
1000000
1200000
HL30 sample1
HL30 sample11
HL30 sample3
HL30 sample6
HL30 sample8
HL30 sample9
LL30 sample10
LL30 sample12
LL30 sample2
LL30 sample4
LL30 sample5
LL30 sample8
ASV´s
0
500
1000
1500
2000
2500
3000
0
200000
400000
600000
800000
1000000
1200000
Sequences
HL45 sample1
HL45 sample11
HL45 sample3
HL45 sample6
HL45 sample7
HL45 sample9
LL45 sample10
LL45 sample12
LL45 sample2
LL45 sample4
LL45 sample5
LL45 sample7
