# Supplementary material for: Protistan Plankton Responses to Variable Light and Upwelling in the Peruvian Humboldt Current System: Insights Into Community Dynamics Under Environmental Change
Source: Ecol Evol. 2026 Jan 12;16(1):e72827. doi: 10.1002/ece3.72827 (PMC12796512; doi:10.1002/ece3.72827)
Supplement: Supplementary file 5 — File S5: ece372827‐sup‐0005‐FileS5.pdf. [file ECE3-16-e72827-s001.pdf]

sample	value	Status	Mesokosmos	light	time
Low_Light_0._Deep_Water_Day_10	1023	ASV richness	8	low	03/27/20
Low_Light_0._Deep_Water_Day_12	1563	ASV richness	8	low	04/02/20
Low_Light_0._Deep_Water_Day_2	1834	ASV richness	8	low	03/01/20
Low_Light_0._Deep_Water_Day_4	488	ASV richness	8	low	03/07/20
Low_Light_0._Deep_Water_Day_5	899	ASV richness	8	low	03/13/20
Low_Light_0._Deep_Water_Day_7	1657	ASV richness	8	low	03/19/20
Low_Light_15._Deep_Water_Day_10	1164	ASV richness	6	low	03/27/20
Low_Light_15._Deep_Water_Day_12	1400	ASV richness	6	low	04/02/20
Low_Light_15._Deep_Water_Day_2	2296	ASV richness	6	low	03/01/20
Low_Light_15._Deep_Water_Day_4	1199	ASV richness	6	low	03/07/20
Low_Light_15._Deep_Water_Day_5	1286	ASV richness	6	low	03/13/20
Low_Light_15._Deep_Water_Day_8	882	ASV richness	6	low	03/21/20
Low_Light_30._Deep_Water_Day_10	1372	ASV richness	4	low	03/27/20
Low_Light_30._Deep_Water_Day_12	1160	ASV richness	4	low	04/02/20
Low_Light_30._Deep_Water_Day_2	2073	ASV richness	4	low	03/01/20
Low_Light_30._Deep_Water_Day_4	2181	ASV richness	4	low	03/07/20
Low_Light_30._Deep_Water_Day_5	1416	ASV richness	4	low	03/13/20
Low_Light_30._Deep_Water_Day_8	1443	ASV richness	4	low	03/21/20
Low_Light_45._Deep_Water_Day_10	1397	ASV richness	2	low	03/27/20
Low_Light_45._Deep_Water_Day_12	1406	ASV richness	2	low	04/02/20
Low_Light_45._Deep_Water_Day_2	1147	ASV richness	2	low	03/01/20
Low_Light_45._Deep_Water_Day_4	1322	ASV richness	2	low	03/07/20
Low_Light_45._Deep_Water_Day_5	1798	ASV richness	2	low	03/13/20
Low_Light_45._Deep_Water_Day_7	1996	ASV richness	2	low	03/19/20
Low_Light_0._Deep_Water_Day_10	0.42	evenness	8	low	03/27/20
Low_Light_0._Deep_Water_Day_12	0.44	evenness	8	low	04/02/20
Low_Light_0._Deep_Water_Day_2	0.58	evenness	8	low	03/01/20
Low_Light_0._Deep_Water_Day_4	0.59	evenness	8	low	03/07/20
Low_Light_0._Deep_Water_Day_5	0.55	evenness	8	low	03/13/20
Low_Light_0._Deep_Water_Day_7	0.56	evenness	8	low	03/19/20
Low_Light_15._Deep_Water_Day_10	0.52	evenness	6	low	03/27/20
Low_Light_15._Deep_Water_Day_12	0.57	evenness	6	low	04/02/20
Low_Light_15._Deep_Water_Day_2	0.55	evenness	6	low	03/01/20
Low_Light_15._Deep_Water_Day_4	0.57	evenness	6	low	03/07/20
Low_Light_15._Deep_Water_Day_5	0.55	evenness	6	low	03/13/20
Low_Light_15._Deep_Water_Day_8	0.53	evenness	6	low	03/21/20
Low_Light_30._Deep_Water_Day_10	0.57	evenness	4	low	03/27/20
Low_Light_30._Deep_Water_Day_12	0.55	evenness	4	low	04/02/20
Low_Light_30._Deep_Water_Day_2	0.59	evenness	4	low	03/01/20
Low_Light_30._Deep_Water_Day_4	0.62	evenness	4	low	03/07/20
Low_Light_30._Deep_Water_Day_5	0.56	evenness	4	low	03/13/20
Low_Light_30._Deep_Water_Day_8	0.56	evenness	4	low	03/21/20
Low_Light_45._Deep_Water_Day_10	0.47	evenness	2	low	03/27/20
Low_Light_45._Deep_Water_Day_12	0.61	evenness	2	low	04/02/20
Low_Light_45._Deep_Water_Day_2	0.68	evenness	2	low	03/01/20
Low_Light_45._Deep_Water_Day_4	0.62	evenness	2	low	03/07/20
Low_Light_45._Deep_Water_Day_5	0.57	evenness	2	low	03/13/20
Low_Light_45._Deep_Water_Day_7	0.58	evenness	2	low	03/19/20
Low_Light_0._Deep_Water_Day_10	2.92	Shannon Index	8	low	03/27/20
Low_Light_0._Deep_Water_Day_12	3.26	Shannon Index	8	low	04/02/20
Low_Light_0._Deep_Water_Day_2	4.33	Shannon Index	8	low	03/01/20
Low_Light_0._Deep_Water_Day_4	3.64	Shannon Index	8	low	03/07/20

Low_Light_0._Deep_Water_Day_5	3.74	Shannon Index	8	low	03/13/20
Low_Light_0._Deep_Water_Day_7	4.12	Shannon Index	8	low	03/19/20
Low_Light_15._Deep_Water_Day_10	3.7	Shannon Index	6	low	03/27/20
Low_Light_15._Deep_Water_Day_12	4.12	Shannon Index	6	low	04/02/20
Low_Light_15._Deep_Water_Day_2	4.28	Shannon Index	6	low	03/01/20
Low_Light_15._Deep_Water_Day_4	4.06	Shannon Index	6	low	03/07/20
Low_Light_15._Deep_Water_Day_5	3.95	Shannon Index	6	low	03/13/20
Low_Light_15._Deep_Water_Day_8	3.58	Shannon Index	6	low	03/21/20
Low_Light_30._Deep_Water_Day_10	4.13	Shannon Index	4	low	03/27/20
Low_Light_30._Deep_Water_Day_12	3.89	Shannon Index	4	low	04/02/20
Low_Light_30._Deep_Water_Day_2	4.48	Shannon Index	4	low	03/01/20
Low_Light_30._Deep_Water_Day_4	4.77	Shannon Index	4	low	03/07/20
Low_Light_30._Deep_Water_Day_5	4.06	Shannon Index	4	low	03/13/20
Low_Light_30._Deep_Water_Day_8	4.06	Shannon Index	4	low	03/21/20
Low_Light_45._Deep_Water_Day_10	3.43	Shannon Index	2	low	03/27/20
Low_Light_45._Deep_Water_Day_12	4.43	Shannon Index	2	low	04/02/20
Low_Light_45._Deep_Water_Day_2	4.82	Shannon Index	2	low	03/01/20
Low_Light_45._Deep_Water_Day_4	4.49	Shannon Index	2	low	03/07/20
Low_Light_45._Deep_Water_Day_5	4.3	Shannon Index	2	low	03/13/20
Low_Light_45._Deep_Water_Day_7	4.4	Shannon Index	2	low	03/19/20
Low_Light_0._Deep_Water_Day_10	0.84	Simpson Index	8	low	03/27/20
Low_Light_0._Deep_Water_Day_12	0.86	Simpson Index	8	low	04/02/20
Low_Light_0._Deep_Water_Day_2	0.94	Simpson Index	8	low	03/01/20
Low_Light_0._Deep_Water_Day_4	0.92	Simpson Index	8	low	03/07/20
Low_Light_0._Deep_Water_Day_5	0.91	Simpson Index	8	low	03/13/20
Low_Light_0._Deep_Water_Day_7	0.95	Simpson Index	8	low	03/19/20
Low_Light_15._Deep_Water_Day_10	0.93	Simpson Index	6	low	03/27/20
Low_Light_15._Deep_Water_Day_12	0.95	Simpson Index	6	low	04/02/20
Low_Light_15._Deep_Water_Day_2	0.93	Simpson Index	6	low	03/01/20
Low_Light_15._Deep_Water_Day_4	0.94	Simpson Index	6	low	03/07/20
Low_Light_15._Deep_Water_Day_5	0.94	Simpson Index	6	low	03/13/20
Low_Light_15._Deep_Water_Day_8	0.9	Simpson Index	6	low	03/21/20
Low_Light_30._Deep_Water_Day_10	0.95	Simpson Index	4	low	03/27/20
Low_Light_30._Deep_Water_Day_12	0.95	Simpson Index	4	low	04/02/20
Low_Light_30._Deep_Water_Day_2	0.95	Simpson Index	4	low	03/01/20
Low_Light_30._Deep_Water_Day_4	0.97	Simpson Index	4	low	03/07/20
Low_Light_30._Deep_Water_Day_5	0.95	Simpson Index	4	low	03/13/20
Low_Light_30._Deep_Water_Day_8	0.94	Simpson Index	4	low	03/21/20
Low_Light_45._Deep_Water_Day_10	0.9	Simpson Index	2	low	03/27/20
Low_Light_45._Deep_Water_Day_12	0.97	Simpson Index	2	low	04/02/20
Low_Light_45._Deep_Water_Day_2	0.98	Simpson Index	2	low	03/01/20
Low_Light_45._Deep_Water_Day_4	0.96	Simpson Index	2	low	03/07/20
Low_Light_45._Deep_Water_Day_5	0.95	Simpson Index	2	low	03/13/20
Low_Light_45._Deep_Water_Day_7	0.96	Simpson Index	2	low	03/19/20
High_Light_0._Deep_Water_Day_1	1127	ASV richness	1	high	02/28/20
High_Light_0._Deep_Water_Day_11	1427	ASV richness	1	high	03/31/20
High_Light_0._Deep_Water_Day_3	1907	ASV richness	1	high	03/05/20
High_Light_0._Deep_Water_Day_6	1541	ASV richness	1	high	03/15/20
High_Light_0._Deep_Water_Day_7	1437	ASV richness	1	high	03/19/20
High_Light_0._Deep_Water_Day_9	1305	ASV richness	1	high	03/25/20
High_Light_15._Deep_Water_Day_1	1341	ASV richness	7	high	02/28/20
High_Light_15._Deep_Water_Day_11	1661	ASV richness	7	high	03/31/20
High_Light_15._Deep_Water_Day_3	1070	ASV richness	7	high	03/05/20

High_Light_15._Deep_Water_Day_6	1335	ASV richness	7	high	03/15/20
High_Light_15._Deep_Water_Day_8	1364	ASV richness	7	high	03/21/20
High_Light_15._Deep_Water_Day_9	1392	ASV richness	7	high	03/25/20
High_Light_30._Deep_Water_Day_1	1632	ASV richness	3	high	02/28/20
High_Light_30._Deep_Water_Day_11	1945	ASV richness	3	high	03/31/20
High_Light_30._Deep_Water_Day_3	1835	ASV richness	3	high	03/05/20
High_Light_30._Deep_Water_Day_6	2055	ASV richness	3	high	03/15/20
High_Light_30._Deep_Water_Day_8	1276	ASV richness	3	high	03/21/20
High_Light_30._Deep_Water_Day_9	1865	ASV richness	3	high	03/25/20
High_Light_45._Deep_Water_Day_1	904	ASV richness	5	high	02/28/20
High_Light_45._Deep_Water_Day_11	1671	ASV richness	5	high	03/31/20
High_Light_45._Deep_Water_Day_3	1619	ASV richness	5	high	03/05/20
High_Light_45._Deep_Water_Day_6	1791	ASV richness	5	high	03/15/20
High_Light_45._Deep_Water_Day_7	1774	ASV richness	5	high	03/19/20
High_Light_45._Deep_Water_Day_9	2678	ASV richness	5	high	03/25/20
High_Light_0._Deep_Water_Day_1	0.65	evenness	1	high	02/28/20
High_Light_0._Deep_Water_Day_11	0.54	evenness	1	high	03/31/20
High_Light_0._Deep_Water_Day_3	0.65	evenness	1	high	03/05/20
High_Light_0._Deep_Water_Day_6	0.55	evenness	1	high	03/15/20
High_Light_0._Deep_Water_Day_7	0.55	evenness	1	high	03/19/20
High_Light_0._Deep_Water_Day_9	0.51	evenness	1	high	03/25/20
High_Light_15._Deep_Water_Day_1	0.58	evenness	7	high	02/28/20
High_Light_15._Deep_Water_Day_11	0.49	evenness	7	high	03/31/20
High_Light_15._Deep_Water_Day_3	0.63	evenness	7	high	03/05/20
High_Light_15._Deep_Water_Day_6	0.56	evenness	7	high	03/15/20
High_Light_15._Deep_Water_Day_8	0.61	evenness	7	high	03/21/20
High_Light_15._Deep_Water_Day_9	0.58	evenness	7	high	03/25/20
High_Light_30._Deep_Water_Day_1	0.62	evenness	3	high	02/28/20
High_Light_30._Deep_Water_Day_11	0.5	evenness	3	high	03/31/20
High_Light_30._Deep_Water_Day_3	0.61	evenness	3	high	03/05/20
High_Light_30._Deep_Water_Day_6	0.64	evenness	3	high	03/15/20
High_Light_30._Deep_Water_Day_8	0.6	evenness	3	high	03/21/20
High_Light_30._Deep_Water_Day_9	0.54	evenness	3	high	03/25/20
High_Light_45._Deep_Water_Day_1	0.62	evenness	5	high	02/28/20
High_Light_45._Deep_Water_Day_11	0.45	evenness	5	high	03/31/20
High_Light_45._Deep_Water_Day_3	0.65	evenness	5	high	03/05/20
High_Light_45._Deep_Water_Day_6	0.64	evenness	5	high	03/15/20
High_Light_45._Deep_Water_Day_7	0.63	evenness	5	high	03/19/20
High_Light_45._Deep_Water_Day_9	0.75	evenness	5	high	03/25/20
High_Light_0._Deep_Water_Day_1	4.59	Shannon Index	1	high	02/28/20
High_Light_0._Deep_Water_Day_11	3.91	Shannon Index	1	high	03/31/20
High_Light_0._Deep_Water_Day_3	4.89	Shannon Index	1	high	03/05/20
High_Light_0._Deep_Water_Day_6	4.03	Shannon Index	1	high	03/15/20
High_Light_0._Deep_Water_Day_7	4.03	Shannon Index	1	high	03/19/20
High_Light_0._Deep_Water_Day_9	3.63	Shannon Index	1	high	03/25/20
High_Light_15._Deep_Water_Day_1	4.18	Shannon Index	7	high	02/28/20
High_Light_15._Deep_Water_Day_11	3.67	Shannon Index	7	high	03/31/20
High_Light_15._Deep_Water_Day_3	4.38	Shannon Index	7	high	03/05/20
High_Light_15._Deep_Water_Day_6	4.06	Shannon Index	7	high	03/15/20
High_Light_15._Deep_Water_Day_8	4.41	Shannon Index	7	high	03/21/20
High_Light_15._Deep_Water_Day_9	4.18	Shannon Index	7	high	03/25/20
High_Light_30._Deep_Water_Day_1	4.59	Shannon Index	3	high	02/28/20
High_Light_30._Deep_Water_Day_11	3.8	Shannon Index	3	high	03/31/20

High_Light_30._Deep_Water_Day_3	4.56	Shannon Index	3	high	03/05/20
High_Light_30._Deep_Water_Day_6	4.92	Shannon Index	3	high	03/15/20
High_Light_30._Deep_Water_Day_8	4.29	Shannon Index	3	high	03/21/20
High_Light_30._Deep_Water_Day_9	4.06	Shannon Index	3	high	03/25/20
High_Light_45._Deep_Water_Day_1	4.23	Shannon Index	5	high	02/28/20
High_Light_45._Deep_Water_Day_11	3.32	Shannon Index	5	high	03/31/20
High_Light_45._Deep_Water_Day_3	4.77	Shannon Index	5	high	03/05/20
High_Light_45._Deep_Water_Day_6	4.77	Shannon Index	5	high	03/15/20
High_Light_45._Deep_Water_Day_7	4.71	Shannon Index	5	high	03/19/20
High_Light_45._Deep_Water_Day_9	5.89	Shannon Index	5	high	03/25/20
High_Light_0._Deep_Water_Day_1	0.97	Simpson Index	1	high	02/28/20
High_Light_0._Deep_Water_Day_11	0.94	Simpson Index	1	high	03/31/20
High_Light_0._Deep_Water_Day_3	0.98	Simpson Index	1	high	03/05/20
High_Light_0._Deep_Water_Day_6	0.92	Simpson Index	1	high	03/15/20
High_Light_0._Deep_Water_Day_7	0.95	Simpson Index	1	high	03/19/20
High_Light_0._Deep_Water_Day_9	0.91	Simpson Index	1	high	03/25/20
High_Light_15._Deep_Water_Day_1	0.94	Simpson Index	7	high	02/28/20
High_Light_15._Deep_Water_Day_11	0.86	Simpson Index	7	high	03/31/20
High_Light_15._Deep_Water_Day_3	0.97	Simpson Index	7	high	03/05/20
High_Light_15._Deep_Water_Day_6	0.94	Simpson Index	7	high	03/15/20
High_Light_15._Deep_Water_Day_8	0.97	Simpson Index	7	high	03/21/20
High_Light_15._Deep_Water_Day_9	0.96	Simpson Index	7	high	03/25/20
High_Light_30._Deep_Water_Day_1	0.97	Simpson Index	3	high	02/28/20
High_Light_30._Deep_Water_Day_11	0.9	Simpson Index	3	high	03/31/20
High_Light_30._Deep_Water_Day_3	0.97	Simpson Index	3	high	03/05/20
High_Light_30._Deep_Water_Day_6	0.98	Simpson Index	3	high	03/15/20
High_Light_30._Deep_Water_Day_8	0.95	Simpson Index	3	high	03/21/20
High_Light_30._Deep_Water_Day_9	0.91	Simpson Index	3	high	03/25/20
High_Light_45._Deep_Water_Day_1	0.96	Simpson Index	5	high	02/28/20
High_Light_45._Deep_Water_Day_11	0.74	Simpson Index	5	high	03/31/20
High_Light_45._Deep_Water_Day_3	0.98	Simpson Index	5	high	03/05/20
High_Light_45._Deep_Water_Day_6	0.98	Simpson Index	5	high	03/15/20
High_Light_45._Deep_Water_Day_7	0.96	Simpson Index	5	high	03/19/20
High_Light_45._Deep_Water_Day_9	0.99	Simpson Index	5	high	03/25/20
